# Design, Radiosynthesis and Preliminary Biological Evaluation in Mice of a Brain-Penetrant ^18^F-Labelled σ_2_ Receptor Ligand

**DOI:** 10.3390/ijms22115447

**Published:** 2021-05-21

**Authors:** Rareş-Petru Moldovan, Daniel Gündel, Rodrigo Teodoro, Friedrich-Alexander Ludwig, Steffen Fischer, Magali Toussaint, Dirk Schepmann, Bernhard Wünsch, Peter Brust, Winnie Deuther-Conrad

**Affiliations:** 1 Department of Neuroradiopharmaceuticals, Research Site Leipzig, Institute of Radiopharmaceutical Cancer Research, Helmholtz-Zentrum Dresden-Rossendorf (HZDR), Permoserstraße 15, 04318 Leipzig, Germany; d.guendel@hzdr.de (D.G.); r.teodoro@hzdr.de (R.T.); f.ludwig@hzdr.de (F.-A.L.); s.fischer@hzdr.de (S.F.); m.toussaint@hzdr.de (M.T.); p.brust@hzdr.de (P.B.); 2 Life Molecular Imaging GmbH, Tegeler Str. 6-7, 13353 Berlin, Germany; 3 Institut für Pharmazeutische und Medizinische Chemie der Westfälischen Wilhelms-Universität Münster (WWU), Corrensstraße 48, 48149 Münster, Germany; dirk.schepmann@uni-muenster.de (D.S.); wuensch@uni-muenster.de (B.W.); 4 The Lübeck Institute of Experimental Dermatology, University Medical Center Schleswig-Holstein, Ratzeburger Allee 160, 23562 Lübeck, Germany

**Keywords:** σ_2_ receptor, transmembrane protein 97, azaindoles, binding affinity, radiochemistry, fluorine-18 labeling, positron emission tomography (PET), brain-penetration, glioblastoma, glioma, F98, rat model, orthotopic

## Abstract

The σ_2_ receptor (transmembrane protein 97), which is involved in cholesterol homeostasis, is of high relevance for neoplastic processes. The upregulated expression of σ_2_ receptors in cancer cells and tissue in combination with the antiproliferative potency of σ_2_ receptor ligands motivates the research in the field of σ_2_ receptors for the diagnosis and therapy of different types of cancer. Starting from the well described 2-(4-(1*H*-indol-1-yl)butyl)-6,7-dimethoxy-1,2,3,4-tetrahydroisoquinoline class of compounds, we synthesized a novel series of fluorinated derivatives bearing the F-atom at the aromatic indole/azaindole subunit. **RM273** (2-[4-(6-fluoro-1*H*-pyrrolo[2,3-b]pyridin-1-yl)butyl]-6,7-dimethoxy-1,2,3,4-tetrahydroisoquinoline) was selected for labelling with ^18^F and evaluation regarding detection of σ_2_ receptors in the brain by positron emission tomography. Initial metabolism and biodistribution studies of [^18^F]**RM273** in healthy mice revealed promising penetration of the radioligand into the brain. Preliminary in vitro autoradiography on brain cryosections of an orthotopic rat glioblastoma model proved the potential of the radioligand to detect the upregulation of σ_2_ receptors in glioblastoma cells compared to healthy brain tissue. The results indicate that the herein developed σ_2_ receptor ligand [^18^F]**RM273** has potential to assess by non-invasive molecular imaging the correlation between the availability of σ_2_ receptors and properties of brain tumors such as tumor proliferation or resistance towards particular therapies.

## 1. Introduction

The presence of two distinct classes of σ receptors, which was postulated already in the 1970s by Martin et al. [1] based on the effects of opiate benzomorphans, was recognized two decades later. Differences in the stereoselectivity, pharmacology, and molecular weight of guinea pig brain σ receptors and the binding sites of σ receptor ligands in PC12 cells, a tumor cell line derived from rat adrenal chromaffin cells, led to the designation of σ_1_ and σ_2_ receptors, respectively [2]. By contrast to the structurally and functionally thoroughly investigated and well described σ_1_ receptor (for recent reviews of structure, function, and therapeutic potential, see, e.g., Tangui [3] or Soriani and Kourrich [4]), a profound characterization of the biology and a comprehensive understanding of the therapeutic potential of the σ_2_ receptor were delayed mainly due to the absent identification of the protein and the coding gene. A significant step forward was the identification of progesterone receptor membrane component 1 (PGRMC1) as part of the protein complex containing the binding site of σ_2_ receptor ligands, which was developed by Mach and colleagues in 2011 [5]. Further detailed analyses of the relation between PGRMC1 and the σ_2_ receptor eventually resulted in the identification of the σ_2_ receptor as the transmembrane protein 97 (TMEM97) by the Kruse lab in 2017, singled out by radioligand binding characteristics from candidate proteins initially isolated via σ_2_ receptor ligand affinity chromatography from calf liver [6]. The suggested involvement of σ receptors [7], TMEM97 [8,9], and PGRMC1 [10] in sterol biosynthesis and availability in cells stimulated the Mach group to perform extensive genome editing studies that not only indicated a link between the σ_2_ receptor (TMEM97) and PGRMC1 with the receptor-mediated internalization of low density lipoprotein (LDL), but suggested the existence of a trimeric transmembrane protein complex consisting of the σ_2_ receptor (TMEM97), PGRMC1, and LDLR1 [11].

The elucidation of the molecular identity of the σ_2_ receptor and the identification of this protein as part of a functional structure regulating the cholesterol homeostasis of cells significantly improved the understanding of the role of the σ_2_ receptor and the therapeutic potential of σ_2_ receptor ligands for neurologic and neurodegenerative disorders as well as cancer (for a most recent summary of the current understanding of this receptor, see, e.g., the Proceedings from the Fourth International Symposium on σ_2_ Receptors [12]).

A much higher expression of σ_2_ receptors in cancer cells in comparison to normal cells revealed by radioligand binding studies in the 1990s [13] led immediately to the evaluation of the potential of σ receptor-targeting radiopharmaceuticals for noninvasive imaging of human malignancies [14] and to the postulation of the potential of σ_2_ receptors as biomarkers of the proliferative status of solid tumors [15]. Besides, particular ligands targeting the σ_2_ receptor have the potential to activate the apoptotic program in cancer cells. The current classification of the σ_2_ receptor ligands as agonists or antagonists is mainly based on this apoptotic potential [16], with pro-apoptotic ligands such as siramesine (Lu 28-179; [17]) designated as agonists. Although the detailed pharmacological mechanism(s) remain to be analyzed, impacts on both ATP-producing pathways [18] and cholesterol homeostasis and metabolism [19] have been discussed as crucial for the anti-cancer effects of σ_2_ receptor ligands.

**Figure 1:**
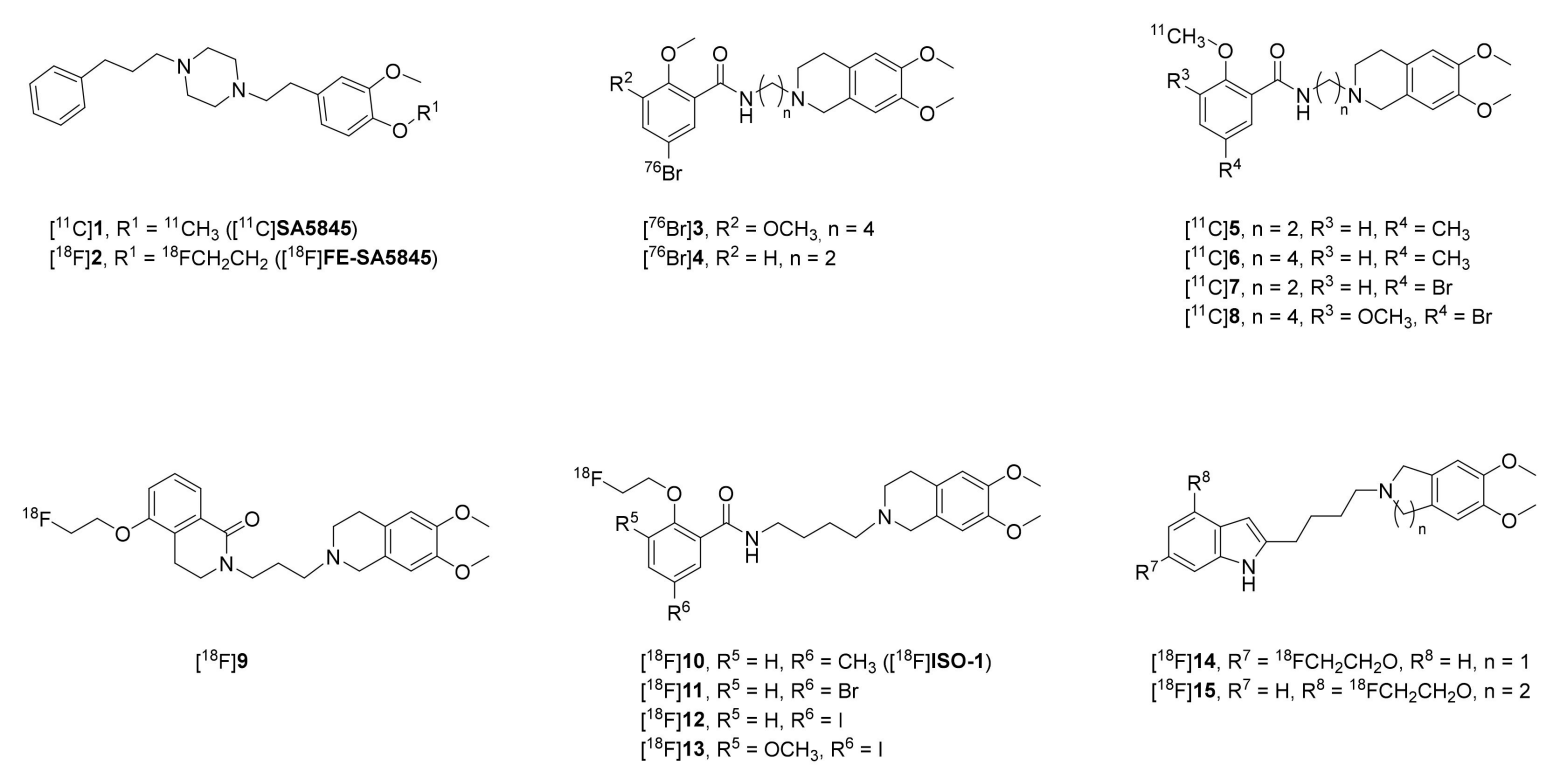
Representative σ_2_ receptor ligands.

While to the best of our knowledge none of the selective agonists of the σ_2_ receptor has been tested in clinical treatment studies, a number of structurally different radiolabeled σ_2_ receptor ligands have been developed and applied as molecular imaging probes in preclinical and clinical positron emission tomography (PET) studies, such as [^11^C]**1**, [^18^F]**2** [20,21], [^76^Br]**3**, [^76^Br]**4** [22], [^11^C]**5**–**8** [23], [^18^F]**9** [24] or [^18^F]**10** [25], called [^18^F]ISO-I and its derivatives [^18^F]**11**, [^18^F]**12**, and [^18^F]**13** (Figure 1) [26]. With the latter radiotracer in hand, remarkable progress was enabled in the research on the correlation between the availability of σ_2_ receptors and tumor-specific processes such as proliferation. As deduced earlier from a substantial and local uptake of [^11^C]RHM-1 in glioblastoma in an orthotopic mouse model, the correlation between the uptake of [^18^F]ISO-I in the tumors of patients with lymphoma, breast cancer, and head and neck cancer and the expression of the proliferation marker Ki-67 [26] supported the suitability of quantitative noninvasive imaging of σ_2_ receptors by PET for monitoring of the proliferative potential of cancer. In addition, a currently recruiting study (NCT03057743) applying PET with [^18^F]ISO-I as baseline scan in patients with metastatic breast cancer is expected to yield data regarding the value of σ_2_ receptor expression as a prognostic biomarker.

The clinically extremely relevant issue of an improvement of the management of patients with brain cancer, however, may not profit from noninvasive imaging with [^18^F]ISO-I due to the limited blood–brain barrier permeability of this particular radiotracer. Thus, we intended to develop a brain-penetrant radiotracer based on the well described ^18^F-fluoroethoxy-substituted 2-(4-(1*H*-indol-1-yl)butyl)-6,7-dimethoxy-1,2,3,4-tetrahydroisoquinoline class of compounds (e.g., compounds [**^18^**F]**14** and [**^18^**F]**15**, Figure 1) recently shown to have a significantly higher brain uptake in comparison to [^18^F]ISO-I (3–4% ID/g at 2 min p.i. [27] vs. 0.76% ID/g at 5 min p.i.; [25]). Using those compounds as a starting point, we intended to further improve the applicability of the corresponding radiotracers for quantitative monitoring of σ_2_ receptors in the brain. To further optimize pharmacological parameters such as selectivity, metabolic stability, and lipophilicity, we synthesized a novel series of nine fluorinated derivatives that all bore the F-atom at the original indole or, as a novel scaffold in the medicinal chemistry of σ_2_ receptor ligands, azaindole (pyrrolopyridine). The azaindole scaffold was introduced to assess the effects of this bioisosteric ring system [28,29]. To explore the potential of azaindole-substituted σ_2_ receptor ligands for in vivo brain imaging, we radiofluorinated **RM273**, the most suitable ligand out of the series developed herein, and examined the pharmacokinetics of [^18^F]**RM273** through dynamic PET studies in mice as well as its binding parameters through brain cryosections of an orthotopic rat glioblastoma model.

## 2. Results and Discussion

### 2.1. Synthesis of Fluorinated Indole and Azaindole Derivatives

**Scheme 1:**
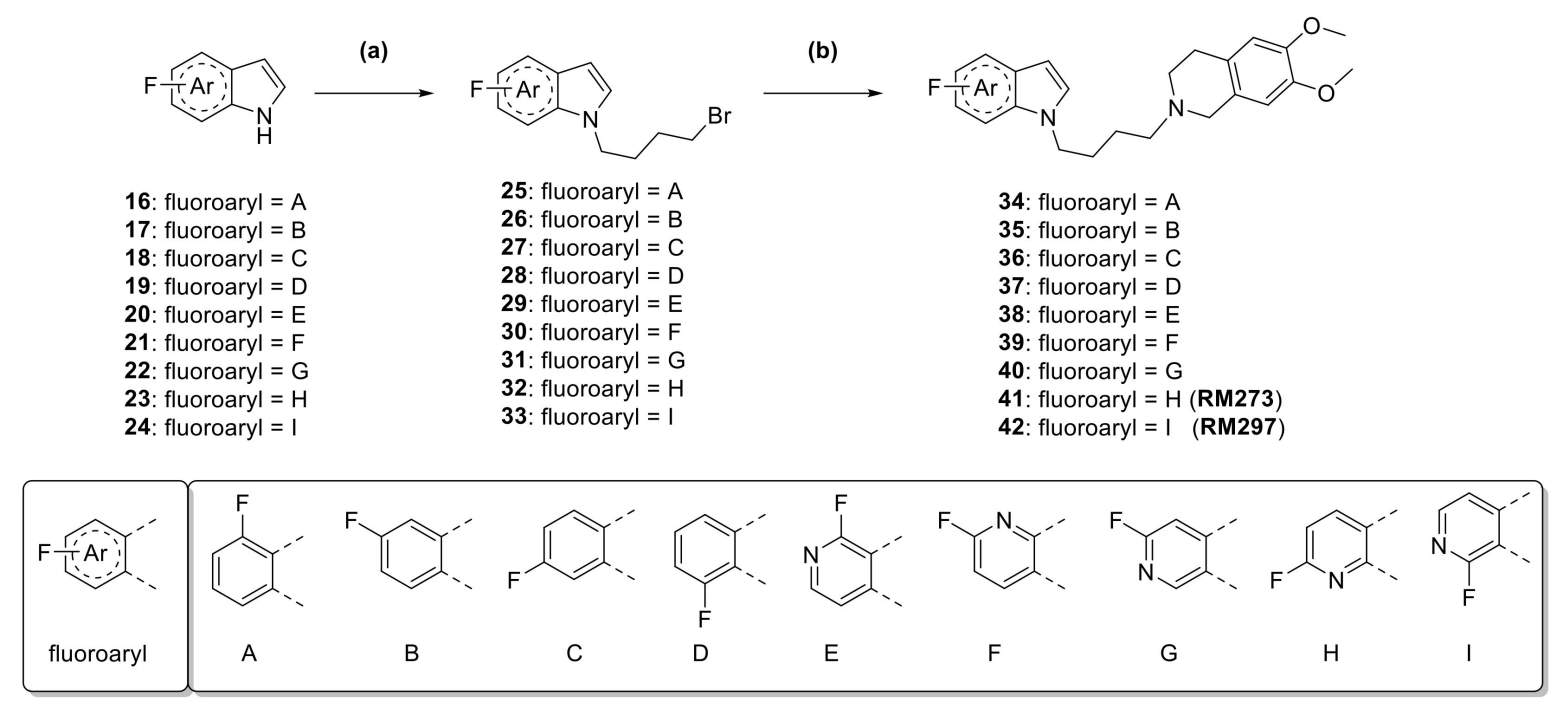
Synthesis of novel fluorinated indole and azaindole derivatives **34**–**42**; reagents and conditions: (**a**) DMF, NaH (60% dispersion in mineral oil, 1.3 eq.), Br(CH_2_)_4_Br (10 eq.), room temperature, 6 h; (**b**) MeCN, 6,7-dimethoxy-1,2,3,4-tetrahydroisoquinoline (**43**, 1.3 eq.), K_2_CO_3_ (3 eq.), 90 °C, 16 h. Abbreviations: DMF *N*,*N*-dimethylformamide; NaH sodium hydride; MeCN acetonitrile; K_2_CO_3_ potassium carbonate.

The synthesis of the newly developed fluorinated derivatives was performed as described previously for similar compounds with minor modifications [27]. First, the commercially available fluorinated indoles and azaindoles were deprotonated with sodium hydride and then treated with 1,4-dibromobutane to give intermediates **25**–**33**, which were further coupled with 6,7-dimethoxy-1,2,3,4-tetrahydroisoquinoline (**43**) under basic reaction conditions to give final compounds **34**–**42** (Scheme 1). Compound **43** was synthesized from 2-(3,4-dimethoxyphenyl)ethan-1-amine and paraformaldehyde [30].

### 2.2. Determination of the In Vitro Affinity

Initially, we assessed the affinity of all derivatives towards the target protein and investigated the inhibition of the binding of [^3^H]DTG in preparations of membrane homogenates obtained from rat liver, which is known to express high levels of σ_2_ receptors [31]. According to the *K*_i_ values summarized in Table 1, all derivatives possess low-nanomolar affinity towards σ_2_ receptors (*K*_i_ values ≤ 14 nM) independent from the particular position of the F-atom at the six-membered ring of the respective bicyclic structure. However, the threefold increase in the affinity confirmed our consideration to further optimize the selectivity of fluorine-substituted indole-based σ_2_ receptor ligands by replacing the indole with azaindole (*K*_i_ values in the range of 7–14 nM vs. 1–6 nM, resp.), irrespective of the particular position of the pyridine-*N*-atom.

To decide which of the nine compounds would be suitable for subsequent radiofluorination, we determined the affinity towards the σ_1_ receptor as the most relevant off-target protein. By competitive radioligand binding studies performed with [^3^H]-(+)-pentazocine, we determined *K*_i_ values in the range of 20–900 nM (Table 1). In contrast to their σ_2_ affinity, the azaindoles **38**–**42** possessed a lower σ_1_ receptor affinity than the indoles **34**–**37**. Furthermore, the positions of both the F- and the N-atom at and within the six-membered ring of the bicyclic structure significantly affected affinity towards the σ_1_ receptor. Among the nine compounds, only the F-substituted 6- and 7-azaindoles bound σ_1_ receptors with an affinity low enough (*K*_i_ values > 500 nM) to exclude confounding binding in imaging studies performed at low- to sub-nanomolar concentration of the radiotracer. To finally select the best derivative for radiofluorination, we calculated the σ_2_:σ_1_ selectivity values and identified **RM273** (**41**) as the most suitable one with a ratio of about 400.

Because ligands targeting σ receptors may be impaired by off-target binding towards the vesicular acetylcholine transporter (VAChT) [31], we determined in the next step the affinity of **RM273** towards this protein. From a *K*_i_ value of >2 μM estimated from the inhibition of the binding of [^3^H]vesamicol in membrane homogenates obtained from PC12 cells stably expressing the rat VAChT, we expected no relevant binding of the corresponding radiofluorinated ligand in PET studies.

Thus, based on radioligand displacement studies indicating sufficiently high affinity towards the σ_2_ receptor and selectivity towards the σ_1_ receptor and VAChT, we decided to develop the radiosynthesis of the azaindole [^18^F]**RM273** ([^18^F]**41**).

### 2.3. Automated Radiosynthesis of [^18^F]**RM273**

**Scheme 2:**
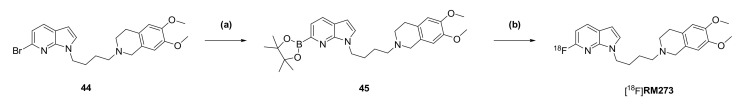
Radiosynthesis of [^18^F]**RM273**; reagents and conditions: (**a**) Pd(dppf)Cl_2_, KOAc, Bis-(pinakolato)-diboron, dioxane, 90 °C, 1 h; (**b**) [^18^F]TBAF, [Cu(OTf)_2_(py)_4_] DMA, *t*-BuOH, 120 °C, 10 min. Abbreviations: [Cu(OTf)_2_(py)_4_] tetrakis(pyridine)copper(II) triflate; Pd(dppf)Cl_2_ [1,1′-bis(diphenylphosphino)ferrocene]dichloro-palladium(II); DMA *N*,*N*-dimethylacetamide; KOAc potassium acetate; TBAF tetrabutylammonium fluoride; *t*-BuOH *tert*-butanol; K_222_ 4,7,13,16,21,24-Hexaoxa-1,10-diazabicyclo[8.8.8]hexacosan.

First attempts to obtain [^18^F]**RM273** via its corresponding bromo-derivative **44** by using either [^18^F]K_222_/K_2_CO_3_ or [^18^F]TBAF failed, leading to the formation of an unidentified product and unreacted [^18^F]fluoride. Hence, a copper-mediated procedure established by the groups of P. J. Scott and V. Gouverneur [33,34,35,36,37] and further developed by several other groups [38,39] was herein enacted to obtain [^18^F]**RM273**. For this purpose, the boronic acid pinacol ester precursor **45** was synthesized in one step from the corresponding bromo-derivative **44** via the Miyaura borylation reaction in high yield (>85%) as shown in Scheme 2 [40].

**Figure 2:**
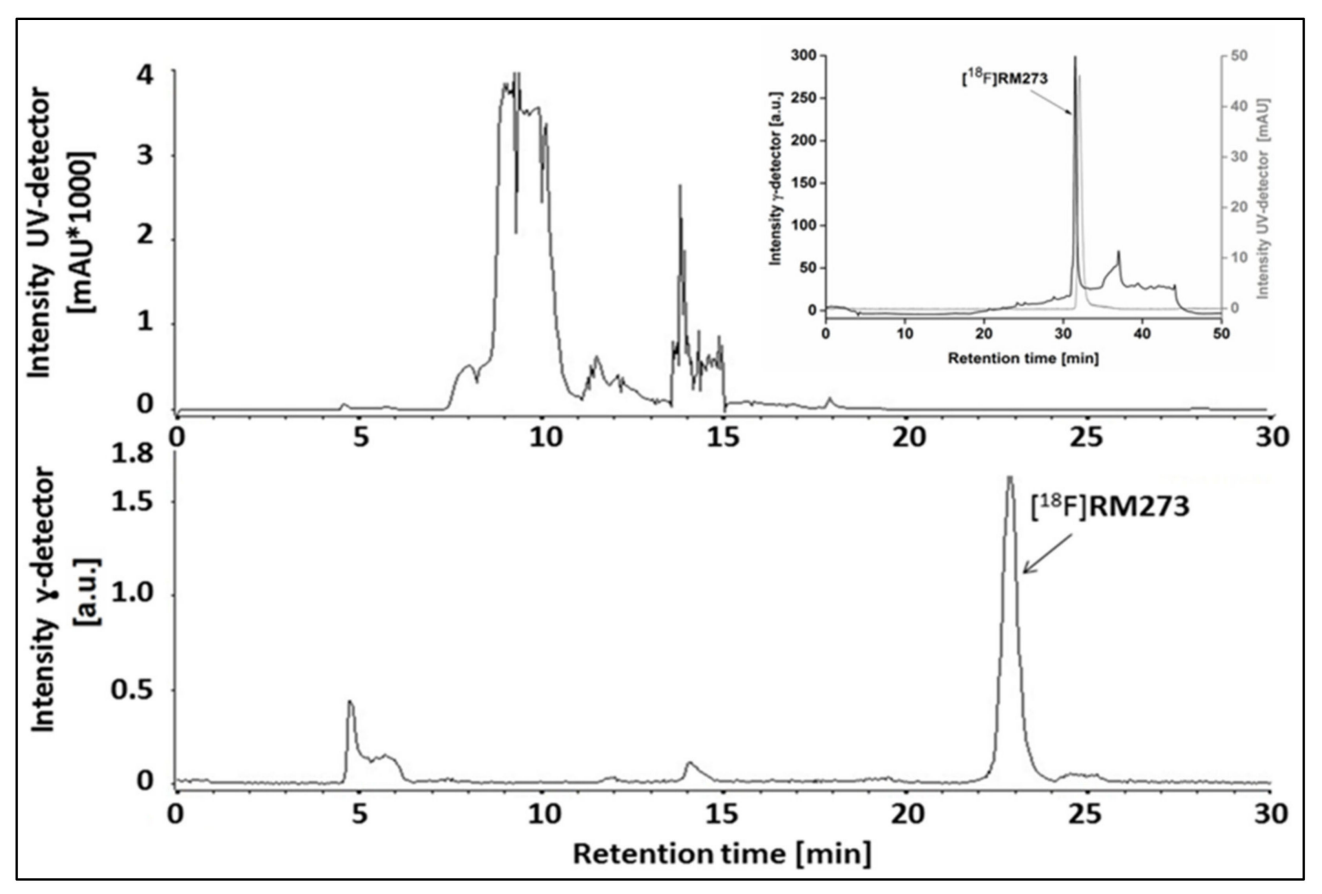
Representative radio- and UV-HPLC chromatograms of [^18^F]**RM273** isolated from the crude reaction mixture via semi-preparative HPLC (Reprosil-Pur C18-AQ column (250 × 10 mm, 5 µm), 58% MeCN/20 mM NH_4_OAc_aq._, flow: 2.2 mL/min), and, in the upper right corner, the radio- (black line) and UV- (gray line) HPLC chromatograms of the identity confirmation of [^18^F]**RM273** co-injected with the reference standard RM273 (Reprosil-Pur C18-AQ column (250 × 4.6 mm; 5 μm); gradient (eluent A 10% MeCN/20 mM NH_4_OAc_aq._; eluent B 90% MeCN/20 mM NH_4_OAc_aq._): 0–10 min 100% A, 10–30 min up to 100% B, 30–35 min 100% B, 35–36 min 100% A, 36–45 min 100% A); flow: 1 mL/min).

As described previously [34,39] and based on our own work [41] on the copper-mediated radiofluorination of aryl boronic acid pinacol esters, manual radiosynthesis of [^18^F]**RM273** (Scheme 2) was performed initially to check if the reaction conditions usually applied for this kind of radiofluorination (e.g., [^18^F]TBAF system in DMA and *t*BuOH as solvents) would give reasonable yields for further translation to an automated radiosynthesis module. Radiochemical yields of about 50% (calculated from the crude reaction mixture, non-decay-corrected) were obtained using the pinalcol precursor **45** and Cu(OTf)_2_(py)_4_ at a molar ratio of approximately 1:4, and no further optimization was carried out. A set of semi-preparative HPLC conditions was tested, and finally optimal conditions (Figure 2) were achieved after the removal of the excess of the copper complex, unreacted [^18^F]fluoride, and polar by-products via an incorporation of an SPE C18 plus cartridge prior to the semi-preparative HPLC isolation of [^18^F]**RM273**.

The manual conditions were transferred to an automated synthesis module (Figure S1, Supporting Information). Particularly for copper-mediated radiofluorinations, this transfer is difficult due to the inert atmosphere in which those automated devices are operated, the usually higher basic environment employed for the elution of [^18^F]fluoride, the order of addition of reagents, and other factors thoroughly explored elsewhere [34,42]. Starting with activities ranging from 2–13 GBq, [^18^F]**RM273** was successfully obtained with a radiochemical yield of 8.1 ± 1.6%, high radiochemical purity (≥99%), and molar activity of about 69–233 GBq/μmol in a total synthesis time of about 60 min (*n* = 3). Analytical radio-HPLC analysis of the final product co-eluted with the corresponding reference compound **RM273** confirmed the identity of the radiotracer (Figure 2).

The in vitro stability of [^18^F]**RM273** was investigated in saline, phosphate-buffered saline (PBS), and TRIS-HCl buffer (50 mM, pH 7.4) at 37 °C for 2 h, and the radioligand remained intact (90%, 94%, and 100%, respectively; saline for 1 h: 95%). The logD_7.4_ of [^18^F]**RM273** was experimentally determined by the shake-flask method as 1.78 ± 0.22 (*n* = 3), which is well within the range of 1–3.5 recommended for brain targeting compounds [43].

### 2.4. In Vitro Evaluation of the σ_2_ Receptor Binding of [^18^F]**RM273** in F98 Rat Glioblastoma

[^18^F]**RM273** with high molar activity and radiochemical purity was used to assess target affinity and selectivity of this azaindole-based σ_2_ receptor ligand in vitro.

**Figure 3:**
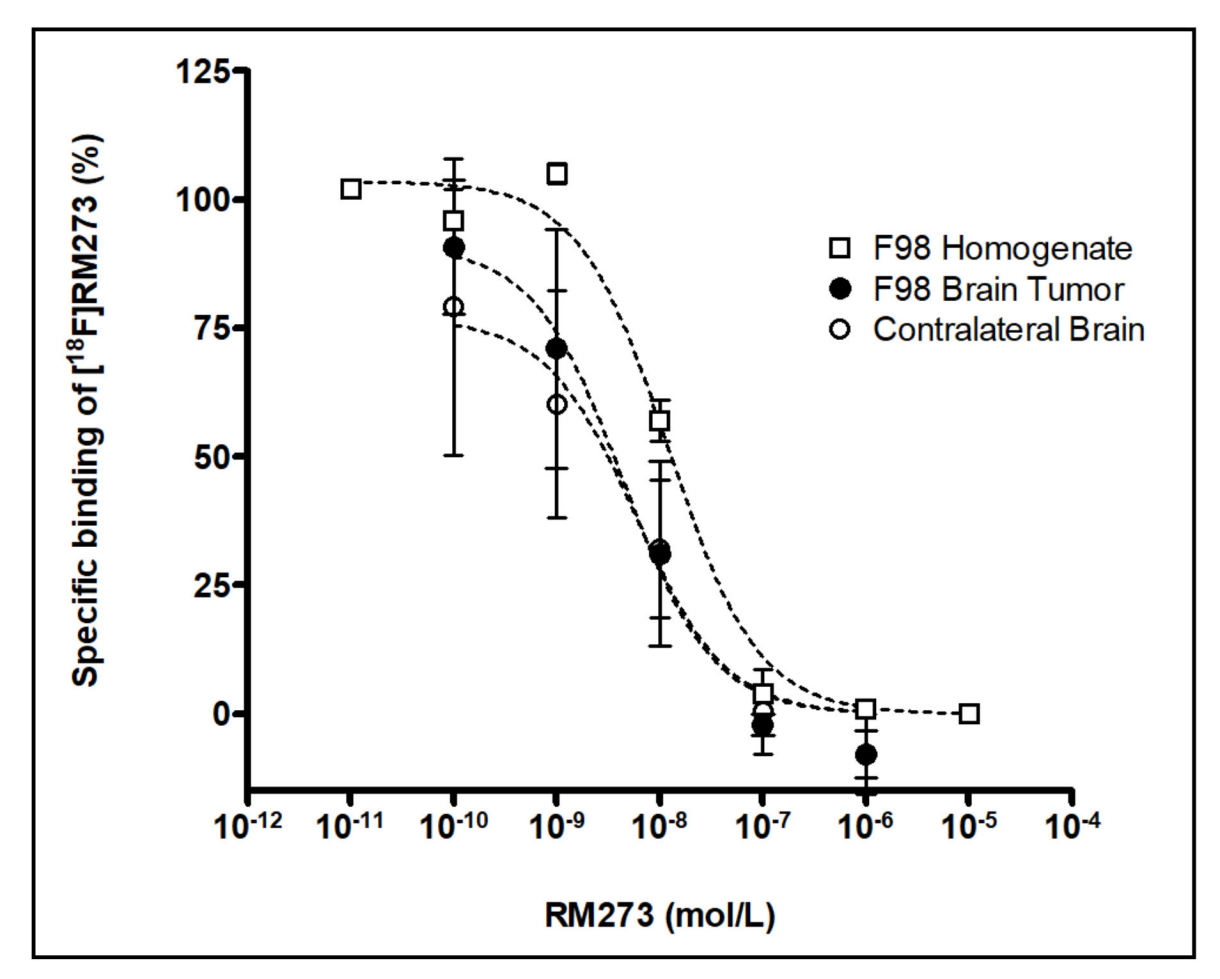
Determination of *K*_D_ and *B*_max_ values of [^18^F]**RM273**. Homologous competition studies were performed by incubation of [^18^F]**RM273** at 5–7 different molar activities either with membrane homogenates of F98 cells (open squares) or cryosections of rat brain (*n* = 2) obtained about three weeks after stereotactic implantation of F98 cells (tumor region = filled circles; contralateral region = open circles). Nonspecific binding was determined by addition of 1 µM ISO-I. The *K*_D_ values were calculated according to the modified Cheng–Prusoff transformation (*K*_D_ = *K*_i_ = IC50—working concentration of the radioligand): *K*_D F98 homogenate_ = 12 nM, *K*_D F98 tumor_ = 4.82 nM, and *K*_D contralateral brain_ = 2.93 nM. For the F98 cell homogenate, the maximum number of binding sites *B*_max_ was calculated by division of the specific binding by the fractional occupancy fp (*B*_max_ = 36,000 fmol/mg protein).

Initially, we investigated the binding of [^18^F]**RM273** on rat brain slices by autoradiography to compare the distribution pattern with that of established σ_2_ receptor ligands and to examine the target specificity. The highest binding was found in the cortical areas and hippocampal structures. It was completely displaceable by the σ_2_ receptor-targeting compounds ISO-I, PB28, and siramesine. Thus, the distribution pattern is comparable to previously reported findings with the established σ_2_ receptor ligands [^3^H]DTG and [^3^H]Lu 28-179 (siramesine) [17,44] and suggests a high specific binding of [^18^F]**RM273** towards σ_2_ receptors. Accordingly, we proceeded to investigate, in a rat glioblastoma model, the potential suitability of [^18^F]**RM273** for molecular characterization of cancer. At first, homologous competition studies using membrane homogenates of the rat glioma cell line F98 were performed to determine the σ_2_ receptor binding parameters of [^18^F]**RM273** cells (Figure 3). Analysis of the inhibition curves provided evidence of a single binding site of [^18^F]**RM273** with a *K*_D_ value of 12 nM and an exceptional receptor density (*B*_max_) of 36,000 fmol/mg protein, equivalent to about 3600 fmol/mg tissue [45]. This result is in line with a previous report on the remarkably high density of σ_2_ receptors in glioblastoma cell lines such as U-138MG or C6 [13].

**Figure 4:**
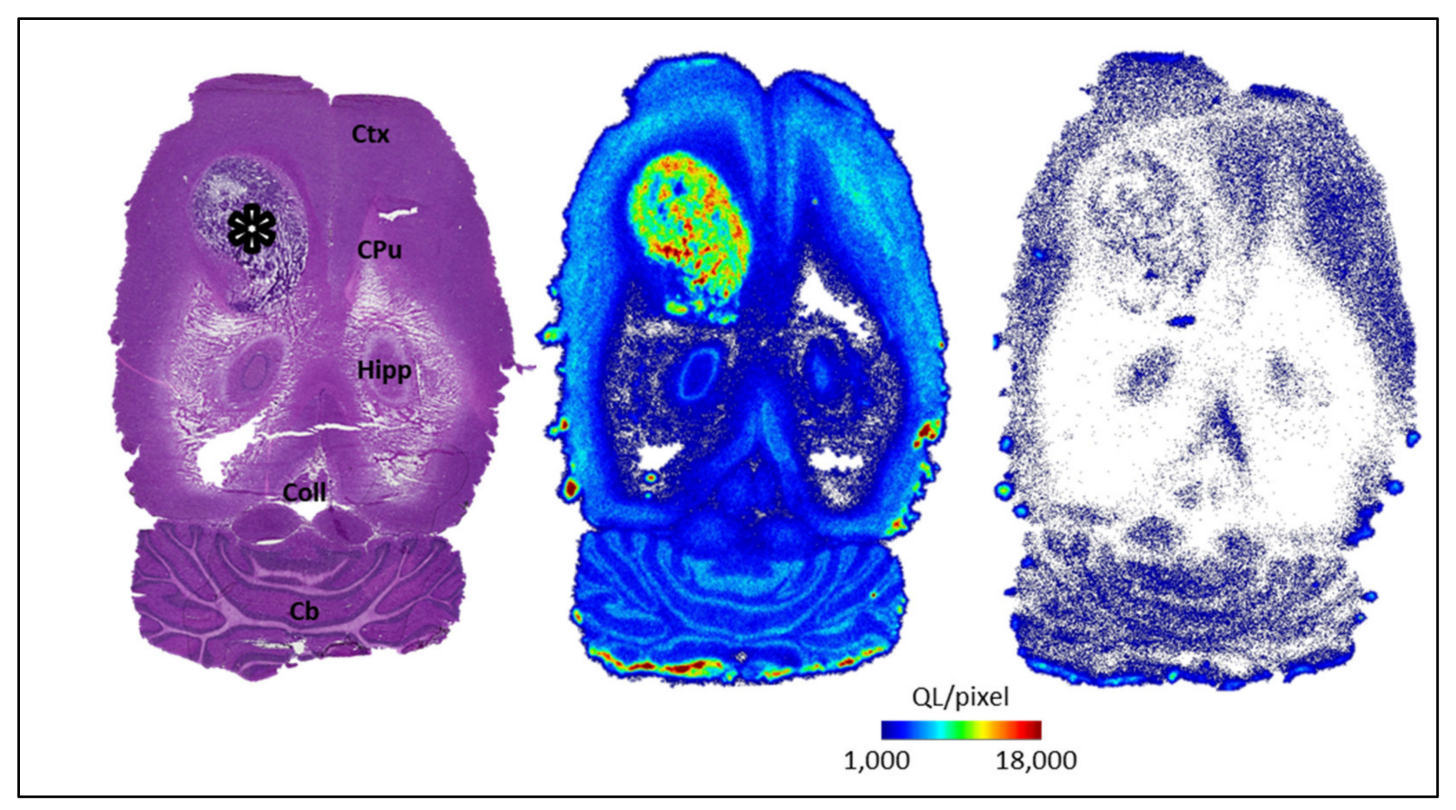
High density of binding sites of [^18^F]**RM273** in the F98 glioblastoma. Incubation of cryosections obtained from rat brain about three weeks after stereotactic implantation of F98 cells in the striatum with 1.5 nM [^18^F]**RM273** without (total binding; middle) or with co-incubation with 1 µM ISO-I (nonspecific binding; right). A remarkably high density of binding sites is detectable in the tumor region (asterisk). The complete displacement of activity also in the tumor region indicates the identity of these binding sites with σ_2_ receptors. For anatomic correlation, Nissl staining was performed (left). Abbreviations: Cb = Cerebellum, Coll = Colliculi, CPu = Caudate putamen, Ctx = Cortex, Hipp = Hippocampus.

We subsequently investigated the binding parameters of [^18^F]**RM273** by in vitro autoradiography using cryosections of rat brains obtained three weeks after stereotactic implantation of F98 rat glioblastoma cells into the left striatum. Initially, we performed a homologous displacement study to directly compare the binding sites of the new radioligand in a healthy brain and a brain tumor. The inhibition curves presented in Figure 3 indicate binding of [^18^F]**RM273** to single binding sites in both brain regions. Furthermore, the affinities of [^18^F]**RM273** in the orthotopically grown F98 tumor and in the contralateral striatum are comparable (*K*_D_ = 4.82 nM vs. 2.93 nM, resp.; *n* = 2) and similar to the values determined in the F98 cell homogenate assay and by the [^3^H]DTG displacement studies. Further supporting the assumption of a tumor-specific overexpression of σ_2_ receptors, a remarkably high density of binding sites of [^18^F]**RM273** in the F98 tumor tissue was observed (Figure 4). Compared to the healthy tissue (contralateral side), about three times more specific binding sites of [^18^F]**RM273** were estimated for the tumor region (32 × 10^−9^ mol/L vs. 94 × 10^−9^ mol/L at 1.5 nM [^18^F]**RM273**).

**Figure 5:**
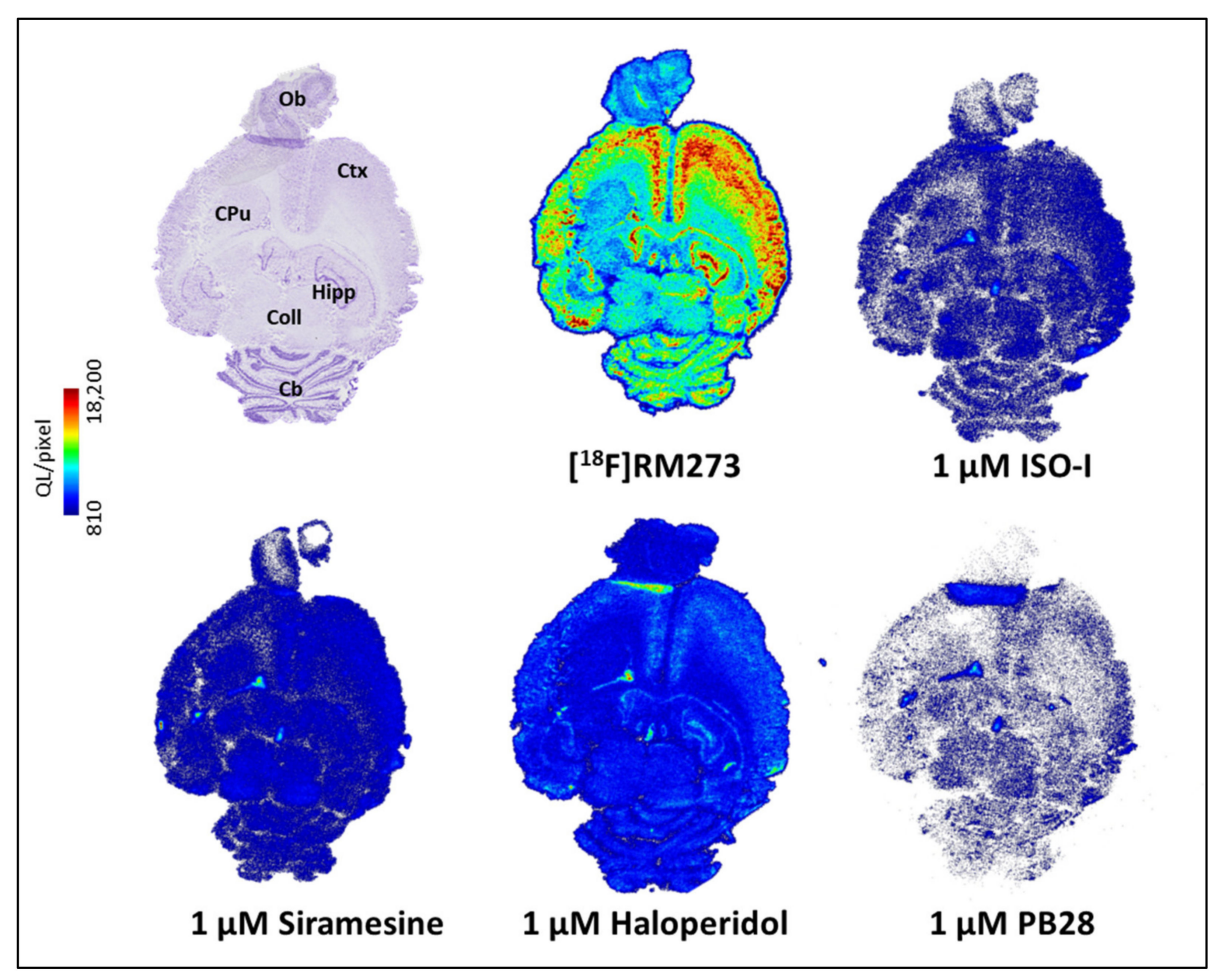
σ_2_ receptor-specific binding of [^18^F]**RM273** in the brain. Incubation of cryosections obtained from rat brain with 0.5 nM [^18^F]**RM273** without (total binding) or with co-incubation with different σ_2_ receptor-targeting ligands at 1 µM (ISO-I, siramesine, haloperidol, PB28). For anatomic correlation, Nissl staining was performed. The activity distribution under baseline conditions ([^18^F]**RM273**) indicates the highest density of binding sites in cortical (Ctx) and hippocampal (Hipp) structures, a medium density in the cerebellum (Cb) and the colliculi (Coll), and a low density in the basal ganglia such as the caudate putamen (CPu).

To confirm the identity of [^18^F]**RM273** binding sites and σ_2_ receptors, we performed additional selectivity studies. The pattern of activity distribution shown in Figure 5 with a high density of binding sites of [^18^F]**RM273** in the hippocampal and cortical structures of the rat brain, medium binding in the diencephalon and the cerebellum, and very low binding in the basal ganglia corresponds very well with the pattern reported for the binding of the established σ_2_ receptor ligands [^3^H]Lu 28-179 (siramesine) and [^3^H]DTG in the rat brain [17,44]. The conclusion that [^18^F]**RM273** binds selectively to σ_2_ receptors in the brain is further supported by the complete displacement of the binding of [^18^F]**RM273** observed by co-incubation with different specific σ_2_ receptor ligands (Figure 5).

Altogether, the autoradiographic studies performed on rat brain in vitro indicate high target selectivity and low nonspecific binding of [^18^F]**RM273**.

### 2.5. PET Imaging of [^18^F]**RM273** in CD1 Mice

To evaluate the potential of [^18^F]**RM273** for non-invasive imaging of σ_2_ receptors in the brain, we administered the radioligand in healthy mice and performed dynamic whole-body PET scans along with complementary ex vivo metabolite analyses.

**Figure 6:**
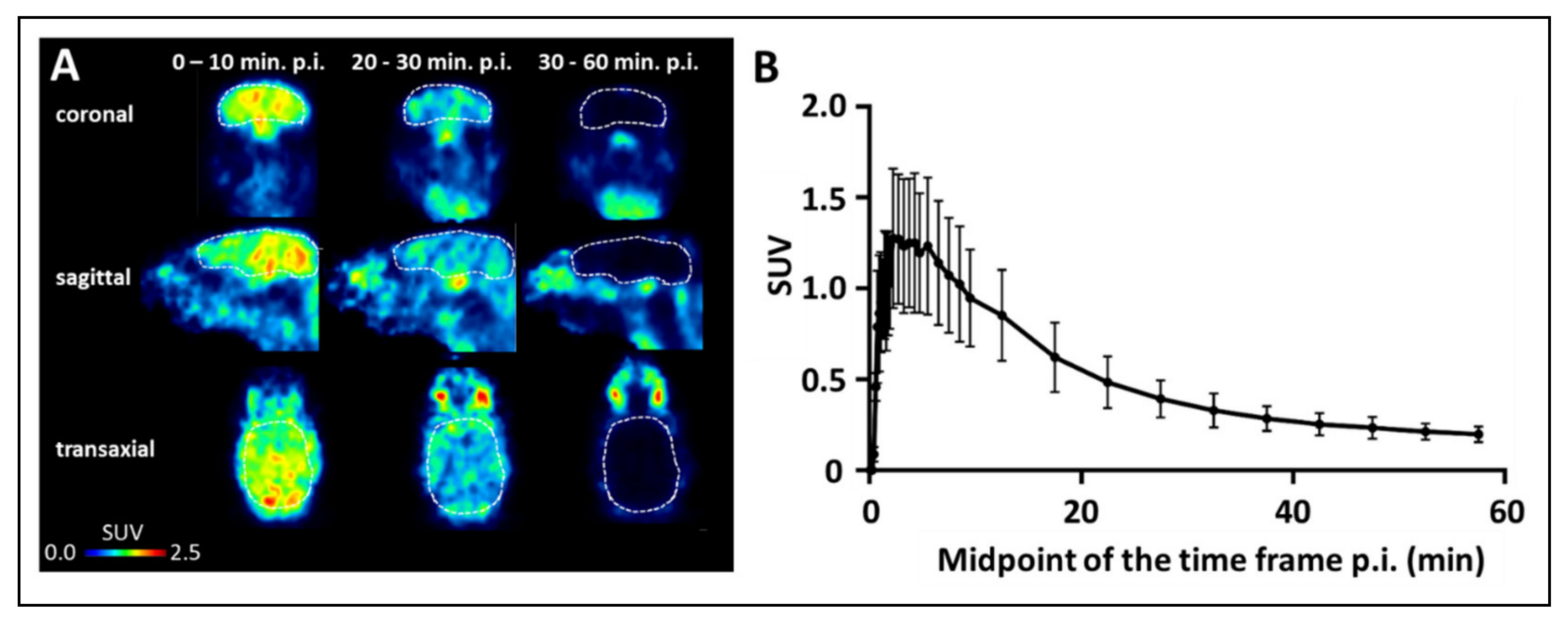
[^18^F]**RM273** is able to penetrate the BBB in healthy mice. (**A**) Representative time-averaged coronal, sagittal, and transaxial PET images of [^18^F]**RM273**. The brain is outlined in white. (**B**) Time–activity curve (mean ± S.D.) of the whole brain region delineated in (**A**) of [^18^F]**RM273** in mice (*n* = 4).

As expected by the optimal lipophilicity of [^18^F]**RM273** (see 2.3, [43]) the PET images and the time–activity curve obtained for the whole brain shown in Figure 6 indicate a fast permeation of the BBB with a mean standardized uptake value (SUV) of 1.3 ± 0.4 at 2.25 min p.i. (*n* = 4). Furthermore, rapid clearance from the brain, with t_1/2_ of 13.1 min after peak time and a peak-to-endpoint ratio of 6.4 ± 0.9, suggests low non-specific binding of [^18^F]**RM273** in vivo.

To further support the interpretation of the PET data, we examined the composition of the radioactive species in the brain at 30 min p.i., assuming that activity accumulated in an organ at this time after bolus administration of a high-affinity radioligand represented mainly binding towards the specific target. From quantitative analysis of the radio-chromatograms, which indicated the presence of solely [^18^F]**RM273** (>99%; *n* = 3; Supplemental Information Table S1 and Figure S2), we concluded that upon the presence of an intact BBB, the PET image derived from [^18^F]**RM273** in the brain was not affected by confounding radiometabolites.

However, some limitations should be noted. First, we were not able to directly compare the regional distribution of [^18^F]**RM273** in rat brain in vitro to the in vivo pattern obtained in mouse brain by PET imaging due to an inadequacy between the size of the mouse brain and the anatomical resolution provided by the available MR system. Therefore, all future studies will be performed in rats to further evaluate the potential of [^18^F]**RM273** to characterize the expression of σ_2_ receptors in brain tumors. Through this approach, the second shortcoming of the current study, limited approval of the target selectivity of [^18^F]**RM273** in vivo, will be addressed as well.

Furthermore, planned in vivo studies performed in a suitable glioblastoma model such as the herein in vitro applied F98 rat model will increase the significance of our preclinical neuro-oncological research. The general suitability of σ2 receptors as imaging biomarkers for the proliferative status of extra-cerebral tumors is impressively reflected by the translation of [^18^F]ISO-I PET from animal models to patient studies [26,46]. A corresponding brain-targeted radioligand may help obtain insights on the relevance of the quantification of σ2 receptors for the diagnosis and therapy of patients with malignancies in the brain.

## 3. Summary

To improve the properties of the well described 2-(4-(1*H*-indol-1-yl)butyl)-6,7-dimethoxy-1,2,3,4-tetrahydroisoquinoline-based σ_2_ receptor ligands for the non-invasive imaging of σ_2_ receptors in the brain, we replaced the indole with an azaindole ring system and introduced an F-atom at the azaindole subunit. The resulting ligands were characterized by low-nanomolar affinity towards σ_2_ receptors and up to 400-fold selectivity towards σ_1_ receptors. Biological evaluation after radiofluorination of the most promising compound, [^18^F]**RM273**, in mice indicated reasonable uptake in the brain that is not affected by brain-penetrant radiometabolites. Furthermore, [^18^F]**RM273** is suitable to detect σ_2_ receptors expressed in orthotopic rat glioblastoma.

## 4. Conclusions

We herein report the development of the PET radioligand [^18^F]**RM273**, which is suitable to monitor the expression of σ_2_ receptors in the brain. This new radioligand has the potential to support research on the validation of σ_2_ receptor expression as a biomarker of tumor-specific processes such as proliferation in cancers of the central nervous system. In addition, the development of therapies targeting σ_2_ receptors in the brain could be promoted by [^18^F]**RM273** PET.

## 5. Materials and Methods

### 5.1. Chemistry

#### 5.1.1. General Information

All chemicals and reagents were purchased from commercially available sources and used without further purification. Moisture-sensitive reactions were conducted under dry argon with oven-dried glassware and anhydrous solvents. Reaction progress was monitored by thin-layer chromatography (TLC) using Alugram^®^ SIL G/UV_254_ pre-coated plates (Macherey-Nagel; Düren; Germany). Spots were identified by using a UV lamp or by dipping the plates into a potassium permanganate solution (3 g KMnO_4_, 20 g K_2_CO_3_, 0.25 mL glacial acid, 300 mL water). For purification of products, flash column chromatography was used with silica gel 40–63 μm from VWR International Chemicals (Darmstadt; Germany). The purity of all the tested compounds was ≥95% as determined by an LC-MS system including a DAD detector (Dionex Ultimate 3000 system incorporating an LPG-3400SD pump, a WPS-3000 TSL autosampler, a TCC-3000SD column compartment, a DAD 3000RS diode array detector and an MSQ Plus low-resolution mass spectrometer (Thermo Fisher Scientific Inc.; Waltham, MA, USA), column: Reprosil-Pur Basic HD (150 × 3 mm; 3 µm; Dr. Maisch GmbH; Ammerbuch; Germany), gradient: 10–90–10% MeCN/20 mM NH_4_OAc_aq._ (*v*/*v*), run time: 15 min, flow rate: 0.6 mL/min, UV-detection: 254 nm). ^1^H-, ^13^C-, and ^19^F-NMR spectra were recorded on VARIAN Mercury plus (300 MHz for ^1^H-NMR, 75 MHz for ^13^C-NMR, 282 MHz for ^19^F-NMR) and BRUKER DRX-400 (400 MHz for ^1^H-NMR, 100 MHz for ^13^C-NMR, 377 MHz for ^19^F-NMR); chemical shifts (δ) in parts per million (ppm) are related to internal tetramethylsilane and coupling constants (*J*) are given with 0.1 Hz. High-resolution mass spectra (HRFT-MS) were recorded on an FT-ICR APEX II spectrometer (Bruker Daltonics; Bruker Corporation; Billerica, MA, USA) using electrospray ionization (ESI).

#### 5.1.2. Chemical Synthesis

General procedure for the synthesis of compounds **34**–**42** and **44**: NaH (60%, suspension in mineral oil, 1.3 mmol, 1.3 eq.) was added to a solution of the fluorine-bearing pyrrolopyridine (compounds **16**–**24**, 1.0 mmol, 1.0 eq.) in DMF at room temperature, and the mixture was stirred for 30 min, after which 1,4-dibromobutane was added at once (10 mmol, 10 eq.). Stirring was continued at room temperature until complete conversion could be detected by TLC (~6 h). Then, NaHCO_3_ solution (5% in water, 20 mL) was added, and the mixture was extracted twice with 10 mL DCM. The combined organic extracts were dried over MgSO_4_, and the solvent was removed under reduced pressure. The oil obtained (compounds **25**–**33**) was used in the next reaction step without further purification. Five mL of anhydrous MeCN containing the bromo-butyl intermediate (compounds **25**–**33**), 6,7-dimethoxy-1,2,3,4-tetrahydroisoquinoline (**43**, 1.3 mmol, 1.3 eq.) and K_2_CO_3_ (3 mmol, 3 eq.) was added, and the mixture was refluxed overnight, after which sat. NaHCO_3_ solution was added and the whole was extracted with EtOAc. The combined organic extracts were washed with sat. NaCl aqueous solution and dried over Na_2_SO_4_. After removing the solvent in vacuo the products were purified by flash chromatography on silica eluting with MeOH/DCM 0.5 to 9.5.

2-[4-(4-Fluoro-1*H*-indol-1-yl)butyl]-6,7-dimethoxy-1,2,3,4-tetrahydroisoquinoline (**34**, 72% yield). ^1^H NMR (400 MHz, Chloroform*-d*) *δ* (ppm) = 7.18–7.02 (m, 3H), 6.75 (ddd, *J* = 10.4, 7.0, 1.5 Hz, 1H), 6.58 (s, 1H), 6.56 (dd, *J* = 3.2, 0.7 Hz, 1H), 6.48 (s, 1H), 4.20–4.11 (m, 2H), 3.83 (s, 3H), 3.82 (s, 3H), 3.47 (s, 2H), 2.78 (t, *J* = 5.9 Hz, 2H), 2.64 (t, *J* = 5.7 Hz, 2H), 2.53–2.45 (m, 2H), 2.00–1.85 (m, 2H), 1.67–1.52 (m, 2H).^13^C NMR (100 MHz, Chloroform*-d*) *δ* (ppm) = 158.13, 154.86, 147.35 (d, *J* = 24.2 Hz), 138.69 (d, *J* = 11.5 Hz), 127.69, 126.29 (d, *J* = 26.6 Hz), 121.79 (d, *J* = 7.9 Hz), 117.55 (d, *J* = 22.5 Hz), 111.35, 109.46, 105.55 (d, *J* = 3.5 Hz), 104.05, 103.80, 97.08, 57.55, 55.91 (2C), 55.68, 50.99, 46.60, 28.62, 28.05, 24.51. ^19^F NMR (377 MHz, Chloroform-*d*) *δ* (ppm) = −122.16 (m). HRMS (ESI+) *m*/*z* = 383.2120, calcd. 383.2129 for C_23_H_28_FN_2_O_2_ [M + H]^+^.

2-(4-(5-Fluoro-1*H*-indol-1-yl)butyl)-6,7-dimethoxy-1,2,3,4-tetrahydroisoquinoline (**35**, 63% yield). ^1^H NMR (400 MHz, chloroform*-d*) *δ* (ppm) = 7.55 (dd, *J* = 8.7, 5.4 Hz, 1H), 7.11 (d, *J* = 3.2 Hz, 1H), 7.05 (dd, *J* = 10.0, 2.3 Hz, 1H), 6.89 (ddd, *J* = 9.6, 8.6, 2.3 Hz, 1H), 6.61 (s, 1H), 6.53 (s, 1H), 6.51–6.42 (m, 1H), 4.12 (t, *J* = 7.1 Hz, 2H), 3.87 (s, 3H), 3.86 (s, 3H), 3.52 (s, 2H), 2.83 (t, *J* = 5.9 Hz, 2H), 2.68 (t, *J* = 5.9 Hz, 2H), 2.52 (t, *J* = 7.3 Hz, 2H), 1.93 (m, 2H), 1.62 (m, 2H). ^13^C NMR (100 MHz, chloroform*-d*) *δ* (ppm) = 160.87, 158.52, 147.55, 147.23, 135.98 (d, *J* = 12.0 Hz), 128.18 (d, *J* = 3.7 Hz), 126.32 (d, *J* = 34.0 Hz), 125.01, 121.60 (d, *J* = 10.1 Hz), 111.39, 109.49, 107.97 (d, *J* = 24.5 Hz), 101.23, 95.77 (d, *J* = 26.2 Hz), 57.64, 55.93 (2C), 55.74, 51.06, 46.43, 28.65, 28.00, 24.58. ^19^F NMR (377 MHz, chloroform*-d*) *δ* (ppm) = −121.29 (m). HRMS (ESI+) *m*/*z* = 383.2125, calcd. 383.2129 for C_23_H_28_FN_2_O_2_ [M + H]^+^.

2-(4-(6-Fluoro-1*H*-indol-1-yl)butyl)-6,7-dimethoxy-1,2,3,4-tetrahydroisoquinoline (**36**, 66% yield). ^1^H NMR (400 MHz, chloroform*-d*) *δ* (ppm) = 7.17 (m, 2H), 7.05 (d, *J* = 3.1 Hz, 1H), 6.84 (td, *J* = 9.1, 2.6 Hz, 1H), 6.50 (s, 1H), 6.40 (s, 1H), 6.35 (d, *J* = 3.2 Hz, 1H), 4.05 (t, *J* = 7.0 Hz, 2H), 3.75 (S, 3H), 3.74 (S, 3H), 3.40 (s, 2H), 2.70 (t, *J* = 6.0 Hz, 2H), 2.56 (t, *J* = 5.9 Hz, 2H), 2.40 (t, *J* = 7.3 Hz, 2H), 1.83 (m, 2H), 1.50 (m, 2H). ^13^C NMR (100 MHz, chloroform*-d*) *δ* (ppm) = 158.90, 156.58, 147.39 (d, *J* = 32.2 Hz), 132.62, 129.35, 128.77 (d, *J* = 10.0 Hz), 126.49, 126.14, 111.38, 110.02, 109.89 (d, *J* = 6.5 Hz), 109.48, 105.56 (d, *J* = 23.3 Hz), 100.93 (d, *J* = 4.7 Hz), 57.58, 55.93 (2C), 55.72, 51.01, 46.55, 28.64, 28.10, 24.57. ^19^F NMR (377 MHz, chloroform*-d*) *δ* (ppm) = −125.76 (m). HRMS (ESI+) *m*/*z* = 383.2132, calcd. 383.2129 for C_23_H_28_FN_2_O_2_ [M + H]^+^.

2-(4-(7-Fluoro-1*H*-indol-1-yl)butyl)-6,7-dimethoxy-1,2,3,4-tetrahydroisoquinoline (**37**, 55% yield). ^1^H NMR (400 MHz, chloroform*-d*) *δ* (ppm) = 7.36 (d, *J* = 7.8 Hz, 1H), 7.06 (d, *J* = 3.1 Hz, 1H), 6.97 (dt, *J* = 7.9, 4.0 Hz, 1H), 6.86 (dd, *J* = 12.9, 7.7 Hz, 1H), 6.58 (s, 1H), 6.54–6.43 (m, 2H), 4.32 (t, *J* = 7.1 Hz, 2H), 3.84 (s, 3H), 3.83 (s, 2H) 3.50 (s, 2H), 2.80 (t, *J* = 5.9 Hz, 2H), 2.66 (t, *J* = 5.9 Hz, 2H), 2.50 (t, *J* = 7.5 Hz, 2H), 1.91 (m, 2H), 1.61 (m, 2H). ^13^C NMR (100 MHz, chloroform*-d*) *δ* (ppm) = 151.40, 148.98, 147.35 (d, *J* = 31.5 Hz), 132.77 (d, *J* = 5.8 Hz), 129.47, 126.55, 126.18, 123.86 (d, *J* = 9.7 Hz), 119.42 (d, *J* = 6.6 Hz), 116.69 (d, *J* = 3.5 Hz), 111.35, 109.48, 107.03 (d, *J* = 18.3 Hz), 101.79 (d, *J* = 1.8 Hz), 57.76, 55.92, 55.72, 51.04, 48.89 (2C), 29.62, 28.66, 24.41. ^19^F NMR (377 MHz, chloroform*-d*) *δ* (ppm) = -135.66 (d, *J* = 12.4 Hz). HRMS (ESI+) *m*/*z* = 383.2129, calcd. 383.2129 for C_23_H_28_FN_2_O_2_ [M + H]^+^.

2-[4-(4-Fluor-1*H*-pyrrolo[3,2-*c*]pyridin-1-yl)butyl]-6,7-dimethoxy-1,2,3,4-tetrahydroisochinolin (**38**, 62% yield). ^1^H NMR (400 MHz, chloroform*-d*) δ 7.81 (dd, *J* = 5.9, 1.3 Hz, 1H), 7.17–7.05 (m, 2H), 6.68–6.53 (m, 2H), 6.47 (s, 1H), 4.16 (t, *J* = 7.0 Hz, 2H), 3.82 (s, 3H), 3.82 (s, 3H), 3.47 (s, 2H), 2.78 (t, *J* = 5.9 Hz, 2H), 2.63 (t, *J* = 5.9 Hz, 2H), 2.49 (t, *J* = 7.2 Hz, 2H), 1.92 (dq, *J* = 9.6, 7.1 Hz, 2H), 1.64–1.47 (m, 2H). ^13^C NMR (101 MHz, chloroform*-d*) δ 158.40, 156.03, 147.50, 147.17, 143.80 (d, *J* = 12.6 Hz), 137.28 (d, *J* = 16.3 Hz), 128.54 (d, *J* = 1.6 Hz), 126.33, 126.04, 111.26, 110.93, 109.33, 103.90 (d, *J* = 4.9 Hz), 99.02 (d, *J* = 5.2 Hz), 57.42, 55.91, 55.87, 55.74, 51.00, 46.89, 28.63, 28.15, 24.39. ^19^F NMR (376 MHz, chloroform*-d*) δ −72.26 (m, 1F). HRMS (ESI+) *m*/*z* = 384.2073, calcd. 384.2081 for C_23_H_27_FN_3_O_2_ [M + H]^+^.

2-[4-(5-Fluor-1*H*-pyrrolo[3,2-*b*]pyridin-1-yl)butyl]-6,7-dimethoxy-1,2,3,4-tetrahydroisochinolin (**39**, 67% yield). ^1^H NMR (300 MHz, chloroform*-d*) δ 7.68 (ddd, *J* = 8.7, 7.0, 0.8 Hz, 1H), 7.33 (d, *J* = 3.2 Hz, 1H), 6.78–6.64 (m, 1H), 6.64–6.52 (m, 2H), 6.47 (s, 1H), 4.15 (t, *J* = 7.0 Hz, 2H), 3.83 (s, 3H), 3.82 (s, 3H), 3.46 (s, 2H), 2.77 (t, *J* = 5.9 Hz, 2H), 2.62 (t, *J* = 5.7 Hz, 2H), 2.48 (t, *J* = 7.2 Hz, 2H), 1.92 (dq, *J* = 9.1, 7.1 Hz, 2H), 1.56 (tt, *J* = 8.0, 6.2 Hz, 2H). ^13^C NMR (75 MHz, chloroform*-d*) δ 160.72, 157.65, 147.55, 147.23, 142.99 (d, *J* = 18.7 Hz), 131.73 (d, *J* = 1.2 Hz), 127.12 (d, *J* = 2.3 Hz), 126.42, 126.09, 121.32 (d, *J* = 10.0 Hz), 111.36, 109.43, 102.11 (d, *J* = 42.8 Hz), 101.62, 57.41, 55.91, 55.90, 55.73, 51.01, 47.07, 28.64, 28.20, 24.42. ^19^F NMR (282 MHz, chloroform*-d*) δ −75.70 (dd, *J* = 7.1, 1.8 Hz, 1F). HRMS (ESI+) *m*/*z* = 384.2079, calcd. 384.2081 for C_23_H_27_FN_3_O_2_ [M + H]^+^.

2-[4-(5-Fluor-1*H*-pyrrolo[2,3-*c*]pyridin-1-yl)butyl]-6,7-dimethoxy-1,2,3,4-tetrahydroisochinolin (**40**, 58% yield). ^1^H NMR (400 MHz, chloroform-*d*) δ 8.33 (s, 1H), 7.33 (d, *J* = 3.1 Hz, 1H), 7.15–6.96 (m, 1H), 6.60 (s, 1H), 6.56–6.43 (m, 2H), 4.25 (t, *J* = 7.0 Hz, 2H), 3.85 (s, 3H), 3.84 (s, 3H), 3.57 (s, 2H), 2.84 (t, *J* = 5.9 Hz, 2H), 2.73 (t, *J* = 5.9 Hz, 2H), 2.56 (t, *J* = 7.3 Hz, 2H), 1.99 (p, *J* = 7.2 Hz, 2H), 1.65 (p, *J* = 7.5 Hz, 2H). ^13^C NMR (101 MHz, chloroform-*d*) δ 147.73, 147.38, 138.00 (d, *J* = 9.2 Hz), 134.13, 132.01, 128.68, 128.49, 125.78, 111.38, 109.45, 100.64 (d, *J* = 6.1 Hz), 97.98 (d, *J* = 40.7 Hz), 57.20, 55.96, 55.92, 55.44, 50.88, 46.90, 29.70, 28.20, 24.30. ^19^F NMR (377 MHz, chloroform-*d*) δ −84.70. HRMS (ESI+) *m*/*z* = 384.2078, calcd. 384.2081 for C_23_H_27_FN_3_O_2_ [M + H]^+^.

2-(4-(6-Fluoro-1*H*-pyrrolo[2,3-b]pyridin-1-yl)butyl)-6,7-dimethoxy-1,2,3,4-tetrahydroisoquinoline (**41**, **RM273**, 67% yield). ^1^H NMR (400 MHz, chloroform*-d*) *δ* (ppm) = 7.92 (dd, *J* = 8.3, 7.8 Hz, 1H), 7.15 (d, *J* = 3.5 Hz, 1H), 6.68 (dd, *J* = 8.3, 1.1 Hz, 1H), 6.57 (s, 1H), 6.49 (s, 1H), 6.45 (d, *J* = 3.5 Hz, 1H), 4.25 (t, *J* = 7.1 Hz, 2H), 3.83 (s, 4H), 3.82 (s, 3H), 3.50 (s, 2H), 2.79 (t, *J* = 5.9 Hz, 2H), 2.66 (t, *J* = 5.9 Hz, 2H), 2.58–2.45 (m, 2H), 1.92 (m, 2H), 1.60 (m, 2H). ^13^C NMR (100 MHz, chloroform*-d*) *δ* (ppm) = 161.19, 158.85, 147.33 (d, *J* = 31.3 Hz), 144.22 (d, *J* = 18.0 Hz), 133.08 (d, *J* = 9.5 Hz), 127.27 (d, *J* = 4.5 Hz), 126.48, 126.11, 117.91 (d, *J* = 2.8 Hz), 111.33, 109.45, 100.99 (d, *J* = 39.0 Hz), 99.95, 57.62, 55.89 (2C), 55.65, 50.99, 44.46, 28.57, 28.26, 24.37. ^19^F NMR (377 MHz, chloroform*-d*) *δ* (ppm) = −75.60 (d, *J* = 7.7 Hz). HRMS (ESI+) *m*/*z* = 384.2074, calcd. 384.2081 for C_23_H_27_FN_3_O_2_ [M + H]^+^.

2-[4-(7-Fluor-1*H*-pyrrolo[2,3-*c*]pyridin-1-yl)butyl]-6,7-dimethoxy-1,2,3,4-tetrahydroisochinolin (**42**, 69% yield). ^1^H NMR (400 MHz, chloroform-*d*) δ 7.72 (dd, *J* = 5.5, 2.0 Hz, 1H), 7.37 (dd, *J* = 5.5, 3.5 Hz, 1H), 7.25 (d, *J* = 3.1 Hz, 1H), 6.59 (s, 1H), 6.56–6.37 (m, 2H), 4.37 (t, *J* = 7.1 Hz, 2H), 3.85 (s, 3H), 3.84 (s, 3H), 3.51 (s, 2H), 2.81 (t, *J* = 5.9 Hz, 2H), 2.67 (t, *J* = 5.9 Hz, 2H), 2.51 (t, *J* = 7.3 Hz, 2H), 1.96 (p, *J* = 7.2 Hz, 2H), 1.72–1.53 (m, 2H). ^13^C NMR (101 MHz, chloroform-*d*) δ 151.10, 148.80, 147.52, 147.20, 138.69 (d, *J* = 7.6 Hz), 134.44, 134.30, 132.53, 126.33 (d, *J* = 35.3 Hz), 118.65 (d, *J* = 27.0 Hz), 114.25 (d, *J* = 4.6 Hz), 111.36, 109.46, 101.84. 57.63, 55.94, 55.77, 51.04, 49.02, 48.98, 29.63, 28.68, 24.33. ^19^F NMR (377 MHz, chloroform-*d*) δ −80.63. HRMS (ESI+) *m*/*z* = 384.2083, calcd. 384.2081 for C_23_H_27_FN_3_O_2_ [M + H]^+^.

2-[4-(6-Brom-1*H*-pyrrolo[2,3-*b*]pyridin-1-yl)butyl]-6,7-dimethoxy-1,2,3,4-tetrahydroisochinolin (**44**, 75% yield). ^1^H NMR (400 MHz, chloroform-*d*) δ 7.72 (d, *J* = 8.1 Hz, 1H), 7.23–7.11 (m, 2H), 6.54 (d, *J* = 28.5 Hz, 2H), 6.43 (d, *J* = 3.5 Hz, 1H), 4.30 (t, *J* = 7.1 Hz, 2H), 3.83 (s, 3H), 3.82 (s, 3H), 3.49 (d, *J* = 1.1 Hz, 2H), 2.79 (t, *J* = 5.9 Hz, 2H), 2.66 (t, *J* = 5.9 Hz, 2H), 2.58–2.42 (m, 2H), 2.05–1.85 (m, 2H), 1.59 (tdd, *J* = 9.9, 6.8, 5.7 Hz, 2H). ^13^C NMR (101 MHz, chloroform-*d*) δ 147.49, 147.18, 134.53, 130.76, 127.89, 126.62, 126.19, 119.16, 111.37, 109.51, 99.85, 57.66, 55.92, 55.89, 55.73, 51.06, 44.37, 28.67, 28.25, 24.36.

6,7-Dimethoxy-2-{4-[6-(4,4,5,5-tetramethyl-1,3,2-dioxaborolan-2-yl)-1*H*-pyrrolo[2,3-*b*]pyridin-1-yl]butyl}-1,2,3,4-tetrahydroisoquinoline (**45**). 200 mg (0.4 mmol, 1 eq.) compound **44**, 170 mg (0.6 mmol, 15 eq.) 4,4,4′,4′,5,5,5′,5′-Octamethyl-2,2′-bis(1,3,2- dioxaborolane), 88 mg (0.9 mmol, 2 eq.) potassium acetate and 33 mg (0.04 mmol, 0.1 eq.) [PdCl_2_(dppf)] were placed in a 50 mL round bottom flask and secured three times. 12 mL of 1,4-dioxane was added under argon countercurrent and the mixture was secured three times. The reaction mixture was then refluxed for 30 min. The cooled reaction solution was filtered through Celite and the filter residue was washed with 50 mL of 1,4-dioxane and the filtrate was concentrated to dryness under reduced pressure. The remaining residue was dissolved in 10 mL demineralized H_2_O and washed 2 × 10 mL Et_2_O after which the aqueous phase was concentrated to dryness by rotary-evaporation to give a glassy solid. Addition of 0.5 mL DCM followed by fast evaporation under reduced pressure afforded **45** as fluffy light brown solid in 85% yield. ^1^H NMR (400 MHz, chloroform-*d*) δ 7.94 (d, *J* = 7.8 Hz, 1H), 7.65 (d, *J* = 7.8 Hz, 1H), 7.30 (d, *J* = 3.5 Hz, 1H), 6.56 (s, 1H), 6.51 (d, *J* = 3.5 Hz, 1H), 6.39 (s, 1H), 4.42 (t, *J* = 6.6 Hz, 2H), 4.02 (s, 2H), 3.82 (s, 3H), 3.80 (s, 3H), 3.31 (ddd, *J* = 18.8, 8.7, 5.6 Hz, 4H), 2.98 (t, *J* = 6.2 Hz, 2H), 2.06–1.89 (m, 2H), 1.74 (p, *J* = 6.8 Hz, 2H), 1.27–1.17 (m, 12H). ^13^C NMR (101 MHz, chloroform-*d*) δ 166.44, 148.75, 148.22, 146.22, 129.41, 128.81, 123.03, 122.65, 122.21, 119.68, 111.00, 108.97, 100.60, 83.60 (2C), 54.81, 51.81, 48.89, 42.75, 28.07, 24.82 (4C), 20.56.

### 5.2. Radiochemistry

#### 5.2.1. Automated Radiosynthesis of [^18^F]**RM273**

Remote controlled radiosynthesis was performed using a Synchrom R&D EVO III automated synthesizer (Elysia-Raytest, Straubenhardt, Germany). The setup of the automat is depicted in the supplemental information (Figure S1) along with the reagents and conditions used as described for the manual synthesis (Supplemental Information).

Briefly, [^18^F]fluoride (4–6 GBq) was trapped on a Waters QMA cartridge and eluted with a solution containing 100 μL of TBAHCO_3_ and 30 μL K_2_CO_3_ dissolved in a mixture of H_2_O/MeCN (1:4, *v*/*v*) into the reaction vessel and dried via azeotropic distillation. An additional 1.5 mL of dried MeCN was added. After complete dryness, a solution containing 2 μmol of boronic acid pinacol ester **45** and 7.5 μmol Cu(py)_4_(OTf)_2_ in DMA/*t*BuOH (2:1, *v*/*v*) was added, and the reaction mixture was stirred at 120 °C for 10 min. To remove the excess of copper catalyst and some other non-radioactive impurities, a Sep Pak® C18 Plus cartridge (5 mL EtOH, 60 mL H_2_O, Waters GmbH, Eschborn, Germany) was applied prior to the semi-preparative HPLC isolation of [^18^F]**RM273**. For that purpose, the reaction mixture was diluted with 18 mL H_2_O and the cartridge eluted with 2.5 mL MeCN. After further dilution with 2.5 mL H_2_O, the solution was transferred to the semi-preparative HPLC. [^18^F]**RM273** was collected in the HPLC collection vial containing 40 mL of H_2_O and trapped in the Sep-Pak^®^ C18 light cartridge. The cartridge was washed with 2 mL H_2_O and [^18^F]**RM273** eluted with 1.3 mL EtOH. This ethanolic solution was transferred outside of the shielded cell, the solvent was evaporated at 70 °C in a gentle stream of nitrogen for 5–10 min, and [^18^F]**RM273** was reconstituted in isotonic saline solution for further biological characterization. Total synthesis time was about 60 min.

#### 5.2.2. Quality Control

Radio-TLC was performed on Alugram^®^ SIL G/UV_254_ pre-coated plates (Macherey-Nagel; Düren, Germany) with PE:EA (1:1, *v*/*v*). The plates were exposed to storage phosphor screens (BAS IP MS 2025 E, GE Healthcare Europe GmbH, Freiburg, Germany) and recorded using the Amersham Typhoon RGB Biomolecular Imager (GE Healthcare Life Sciences). Images were quantified with the ImageQuant TL8.1 software (GE Healthcare Life Sciences).

Analytical chromatographic separations were performed on a JASCO LC-2000 system, incorporating a PU-2080*Plus* pump, AS-2055*Plus* auto injector (100 μL sample loop), and a UV-2070*Plus* detector coupled with a γ-detector (GABI Star; raytest Isotopenmessgeräte GmbH, Straubenhardt, Germany). Data analysis was performed with the Galaxie chromatography software (Agilent Technologies, Santa Clara, CA, USA) using chromatograms obtained at 254 nm.

Radiochemical yield, radiochemical purity, and analyses of plasma and brain samples were assessed via reversed-phase HPLC (RP-HPLC) in gradient mode (0–10 min: 10% MeCN/20 mM NH_4_OAc_aq._, 10–30 min: 10%→90% MeCN/20 mM NH_4_OAc_aq._, 30–35 min: 90% MeCN/20 mM NH_4_OAc_aq._, 35–36 min: 90%→10% MeCN/20 mM NH_4_OAc_aq._ 36–45 min: 10% MeCN/20 mM NH_4_OAc_aq._).

Molar activity was determined using analytical radio-HPLC with a Reprosil-Pur C18-AQ column (250 × 4.6 mm, 5 μm) and 42% MeCN/20 mM NH_4_OAc_aq._ as eluent at a flow rate of 1 mL·min^−1^ and UV detection at 208 nm.

#### 5.2.3. Determination of In Vitro Stability

In vitro stability of [^18^F]**RM273** was determined in saline, phosphate-buffered saline (PBS), and TRIS (50 mM, pH 7.4) by incubation of approx. 5 MBq in 500 µL of each medium at 37 °C. Samples were taken after 30, 60, and 120 min and analyzed by analytical radio-HPLC with a gradient system as applied in Section 5.2.2.

#### 5.2.4. Determination of Lipophilicity (LogD_7.4_)

Log*D*_7.4_ of [^18^F]**RM273** was experimentally determined in n-octanol/PBS; 0.01 M, pH 7.4) at room temperature by the shake-flask method. The measurement was performed twice in triplicate [43].

### 5.3. Biological Experiments

All studies involving animals were carried out according to the guidelines of the Declaration of Helsinki, the Directive 2010/63/EU of the European Parliament and of the Council of September 22nd, 2010, on the protection of animals and the German Animal Welfare Act, and were approved by the responsible authorities (Landesdirektion Sachsen; No. DD24.1-5131/446/19, TVV 18/18, 20.06.2018; No. DD25-5131/446/38, TVV 36/18, 03.03.2021).

#### 5.3.1. Determination of Binding Affinities by Homogenate Assays

Binding affinity experiments to assess the *K*_i_ values of test compounds towards rat σ_2_ receptors, human σ_1_ receptors, and rat VAChT were performed according to protocols previously published by our group [47]. The same protocol was used to determine the *K*_D_ and *B*_max_ values of **RM273** towards σ_2_ receptors expressed in F98 cells by homologous competition analyzing the displacement of [^18^F]**RM273** by **RM273**.

#### 5.3.2. In Vitro Autoradiography

In vitro autoradiography was performed using cryosections of brains of a healthy rat (SPRD, female, 250 g; Medizinisch-Experimentelles Zentrum, Universität Leipzig, Leipzig, Germany) and brains obtained from rats (Fisher F344/HanZtmRj, male, ≈260 g; Janvier Labs, Le Genest-Saint-Isle, France) 23 days after stereotactic implantation of 5 × 10^4^ F98 cells in the right striatum (*n* = 2).

Immediately after isolation, the brains were frozen by immersion in isopentane at −25 °C. Cryosections of 12 μm (Microm HM560 cryostat, Thermo Scientific, Schwerte, Germany) were mounted on microscopic glass slides, dried at room temperature, and stored at −25 °C. For the experiment, the slices were dried at room temperature under a stream of cold air before pre-incubation in PBS, pH 7.4, at room temperature for 15 min. After decantation, the slices were dried again under a stream of cold air before incubation with [^18^F]**RM273** (working concentration ≈ 1 nM) without (total binding) or with ISO-I, siramesine, or PB28 (each 10^−6^ M) to determine the nonspecific binding of the radioligand. Co-incubation with serial dilutions of **RM273** (10^−10^–10^−6^ M) was performed to determine an inhibition curve. After 60 min, the incubation at room temperature was terminated by decanting and washing the slides by two consecutive incubations in ice-cold washing buffer (50 mM TRIS-HCl, pH 7.4 at 4 °C) for two min each, followed by dipping in ice-cold demineralized water for 5 sec and drying under a stream of cold air. In parallel, for subsequent quantification of the data obtained from the homologous competition experiment, defined volumes, and thus defined activities of the radioligand solution, were pipetted onto a microscopic glass slide (0.5–3 μL = 0.0355–0.213 kBq) and dried. Afterwards the tissue samples along with the standards were exposed to an ^18^F-sensitive phosphor screen (ABC) for two hours. Image acquisition was performed using a phosphorimager (HD-CR 35; DÜRR NDT GmbH & Co. KG, Bietigheim-Bissingen, Germany). Analysis of the digitized images was performed by image analysis software (AIDA; Elysia-raytest GmbH, Straubenhardt, Germany).

Irregular regions of interest (ROI) were drawn on the standard spots and the different brain regions, which were confirmed by Nissl staining of the identical brain sections. Optical density measured in each ROI was expressed as quantum levels per unit area (QL/pixel) with a pixel size of 12.5 μm^2^. From the calibration curve obtained from the standards by plotting QL vs. activity, the QL values of the ROIs were converted to activity values followed by conversion to chemical concentration of **RM273** based on the molar activity of [^18^F]**RM273**. Region-specific *K*_D_ and *B*_max_ values of **RM273** were estimated from the inhibition curves using the same procedure applied for the homogenate assays.

#### 5.3.3. PET Studies

The biodistribution of [^18^F]**RM273** in mice was assessed by dynamic small animal PET (Nanoscan, Mediso, Budapest, Hungary) 60 min recordings, followed by T1 weighted (GRE, TR/TE = 15.0/2.4 ms, 252/252, FA = 25°) imaging for anatomical correlation and attenuation correction. The mice weighing 31.7 ± 3.8 g were anaesthetized with 2% isoflurane in 60% oxygen and placed on a thermostatically heated animal bed. [^18^F]**RM273** (7.2 ± 1.1 MBq) was injected i.v. into the lateral tail vein (bolus within 5 s) at the start of the PET-acquisition. List mode PET data were binned as a series of attenuation-corrected sinogram frames (12 × 10 s, 6 × 30 s, 5 × 60 s and 10 × 300 s) and were reconstructed by Ordered Subset Expectation Maximization (OSEM3D) with four iterations, six subsets, and a voxel size of 0.4 mm^3^ (Nucline v2.01, Mediso, Hungary). The analysis of reconstructed datasets was performed with PMOD software (v4.103, PMOD Technologies LLC, Zurich, Switzerland). GraphPad Prism (v9, San Diego, CA, USA) was used to calculate the area under the curve (AUC), as well as to determine the peak TAC half-life time by fitting of time–activity curves with dissociation one phase exponential decay setting t_0_ at peak of TAC.

#### 5.3.4. Quantification of Radiometabolites

About 30 MBq [^18^F]**RM273** dissolved in about 150 µL isotonic saline was administered intravenously as a bolus in the tail vein of in awake female CD-1 mice weighing about 33 g (*n* = 3). At 30 min p.i., the animals were anesthetized and blood was withdrawn by retrobulbar bleeding using glass capillaries. Immediately afterwards, the animals were euthanized by cervical dislocation, and released urine sampled. Blood plasma was obtained from the whole blood sample by centrifugation (2 min, 8000 rpm, room temperature). In addition, the brain, the liver, and the pancreas were isolated and homogenized in 1 mL demineralized water on ice (1000 rpm, 10 strokes; glass vessel, PTFE plunger; Potter S, B. Braun Biotech International, Goettingen, Germany). Bone marrow (*n* = 2) was isolated by flushing the femur with about 200 µL isotonic saline.

The samples were further processed for subsequent radio-chromatographic analyses. Two consecutive extractions were performed as duplicate for plasma and brain determinations. Plasma and brain samples were added to an ice-cold MeOH/H_2_O mixture (9:1, *v*/*v*). The samples were vortexed for 3 min, incubated on ice for 5 min, and centrifuged at 12,000 rpm for 5 min. Supernatants were collected, the precipitates were re-dissolved in 100 µL of extraction solvent, and the extraction procedure was repeated. The activities of supernatants and precipitates were measured in a γ-counter (1480 WIZARD, Fa. Perkin Elmer), and the extraction efficiencies were calculated as ratio of the radioactivity in the supernatant to the radioactivity in the original sample (supernatant + precipitate). The supernatants from both extractions were combined, concentrated at 70 °C under argon stream up to a remaining volume of 100 µL, and subsequently analyzed by analytical radio-HPLC with a gradient system as applied in Section 5.2.2.

## Figures and Tables

**Table 1:** In vitro binding affinities of compounds **34**–**42** towards the σ_1_ receptor and the σ_2_ receptor.

	Compound	*K*_i_ σ_1_ (nM)	*K*_i_ σ_2_ (nM)	*Selectivity* (*K*_i_ σ_1_/*K*_i_ σ_2_)
**34**	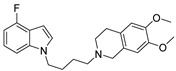	21 ^a^	14	1.5
**35**	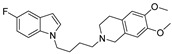	116 ^a^	12	9.7
**36**	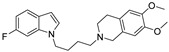	46 ^a^	7.1	6.5
**37**	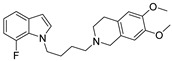	80 ^a^	10	8
**38**	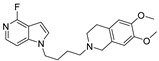	80 ^b^	5.5	14
**39**	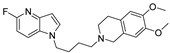	122 ^b^	3.8	32
**40**	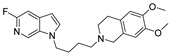	85 ^b^	1.0	85
**41 (RM273)** ^c^	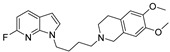	691 ^b^	1.6	432
**42 (RM297)**	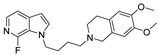	916 ^b^	2.7	339
**ISO-1**		n.d.	10 ^d^	
**Haloperidol**		4.9 ^a,e^	21 ^e^	
**Siramesine**		13 ^a^	30	

n.d. not determined. ^a^ Determined at HZDR Leipzig towards human σ_1_ receptors. ^b^ Determined at WWU towards guinea pig σ_1_ receptors. ^c^ Displacement of [^3^H]vesamicol from rat VAChT: *K*_i_ > 2 µM. ^d^ Reported from another lab: *K*_i_ = 7 nM [32]. ^e^ Taken from previous publication [27].

## Supplementary Materials

The following are available online at https://www.mdpi.com/article/10.3390/ijms22115447/s1.
