# Supplementary material for: Design, Radiosynthesis and Preliminary Biological Evaluation in Mice of a Brain-Penetrant 18F-Labelled σ2 Receptor Ligand
Source: Int J Mol Sci. 2021 May 21;22(11):5447. doi: 10.3390/ijms22115447 (PMC8196714; doi:10.3390/ijms22115447)
Supplement: Supplementary file 1 [file ijms-22-05447-s001.zip › ijms-1214463-supplementary.pdf]

Supplemental Information

Design, radiosynthesis and preliminary biological evaluation in mice of a brain-penetrant ^{18}F-labelled σ_2 receptor ligand

Rareş-Petru Moldovan, Daniel Gündel, Rodrigo Teodoro, Friedrich-Alexander Ludwig, Steffen Fischer, Magali Toussaint, Dirk Schepmann, Bernhard Wünsch, Peter Brust, Winnie Deuther-Conrad

Content:

1. Manual radiosynthesis of [^{18}F]RM273
2. Automated radiosynthesis of [^{18}F]RM273
3. Quantification of radiometabolites in mouse
4. ^{1}H NMR spectra of compounds 34-42, 44 and 45

1. Manual radiosynthesis of [^{18}F]RM273.

No-carrier-added (n.c.a.) [^{18}F]fluoride was produced via the $^{18}O(p,n)^{18}F$ nuclear reaction by irradiation of a [^{18}O]H_2O target (Hyox 18 enriched water; Rotem Industries Ltd, Mishor Yamin, Israel) on a Cyclone®18/9 (IBA RadioPharma Solutions, Louvain-la-Neuve, Belgium) with 18 MeV proton beam using a Nirta® [^{18}F]fluoride XL target or [^{18}O]H_2O recycled by the established in-house method.(34) Starting with 1–2 GBq of n.c.a. [^{18}F]fluoride, [^{18}F]F^--containing anion resin [Sep-Pak® Accell Plus QMA Carbonate Plus light cartridge (Waters GmbH, Eschborn, Germany)] was eluted with a solution composed of 100 µL of tetra-n-butylammonium hydrogen carbonate ($TBAHCO_3$, 0.075 M, ABX advanced biochemical compounds GmbH, Radeberg, Germany) and K_2CO_3 (20 mg/mL solution, 0.6 mg, 4.3 µmol) in H_2O/MeCN (1:4, *v/v*) in a total volume of 1.2 mL. The solution was transferred directly into a 5 mL microwave V-vial (CEM® Corporation, Matthews, NC, USA) and azeotropically dried under vacuum and argon flow in the microwave cavity (Discover PETwave microwave CEM® corporation, 50–60 °C, 75 W) for 8–10 min. Additional aliquots of MeCN (2 × 1.0 mL) were added during the drying process. After complete dryness, a solution containing the pinacol ester precursor **45** (2-5 µmol) and tetrakis(pyridine)copper(II) triflate [$Cu(py)_4(OTf)_2$] (7.5-15 µmol) in *N,N*-dimethylacetamide (DMA, 600 µL) and *tert*-butanol (*tert*-BuOH, 300 µL) was added to the reactive anhydrous [^{18}F]TBAF. The reaction was monitored in different time points (up to 20 min) at 110-130 °C via radio-thin layer chromatography (radio-TLC) and high performance liquid chromatography (HPLC, see quality control section). After the completion of the reaction, the crude reaction mixture was diluted with 18 mL H_2O and passed through a Sep-Pak® C18 Plus cartridge (Waters GmbH, Eschborn, Germany) to remove the excess of $Cu(py)_4(OTf)_2$ and some UV impurities. The cartridge was then eluted with 2.5 mL of MeCN and further diluted with 2.5 mL of H_2O. This solution was directly applied onto a semi-preparative HPLC system composed by a Reprosil-Pur C18-AQ column (Dr. Maisch HPLC GmbH, Germany) with an eluent of 58 % MeCN/20 mM $NH_4OAc_{aq.}$ at a flow rate of 2.2 mL·min^{-1}. [^{18}F]**RM273** was collected and diluted in 40 mL of H_2O. Final purification was performed using a Sep-Pak® C18 Light cartridge (Waters, Milford, MA, USA) followed by elution with 1.3 mL of EtOH. To obtain an injectable solution, the solvent was concentrated under a gentle nitrogen stream at 70 °C and [^{18}F]**RM273** was formulated in a sterile isotonic saline solution (5–10 % EtOH, *v/v*). The identity of [^{18}F]**RM273** was verified by radio-HPLC analysis of an aliquot of the radiotracer solution co-injected with the corresponding reference compound **RM273**. Radiochemical and chemical purities were assessed by radio-TLC and analytical HPLC. Molar activities were determined based on aliquots taken from the formulation, and the mass determination for the corresponding reference standard was performed via a calibration curve obtained under the same analytical HPLC conditions (see quality control section).

2. Automated radiosynthesis of [^{18}F]RM273.

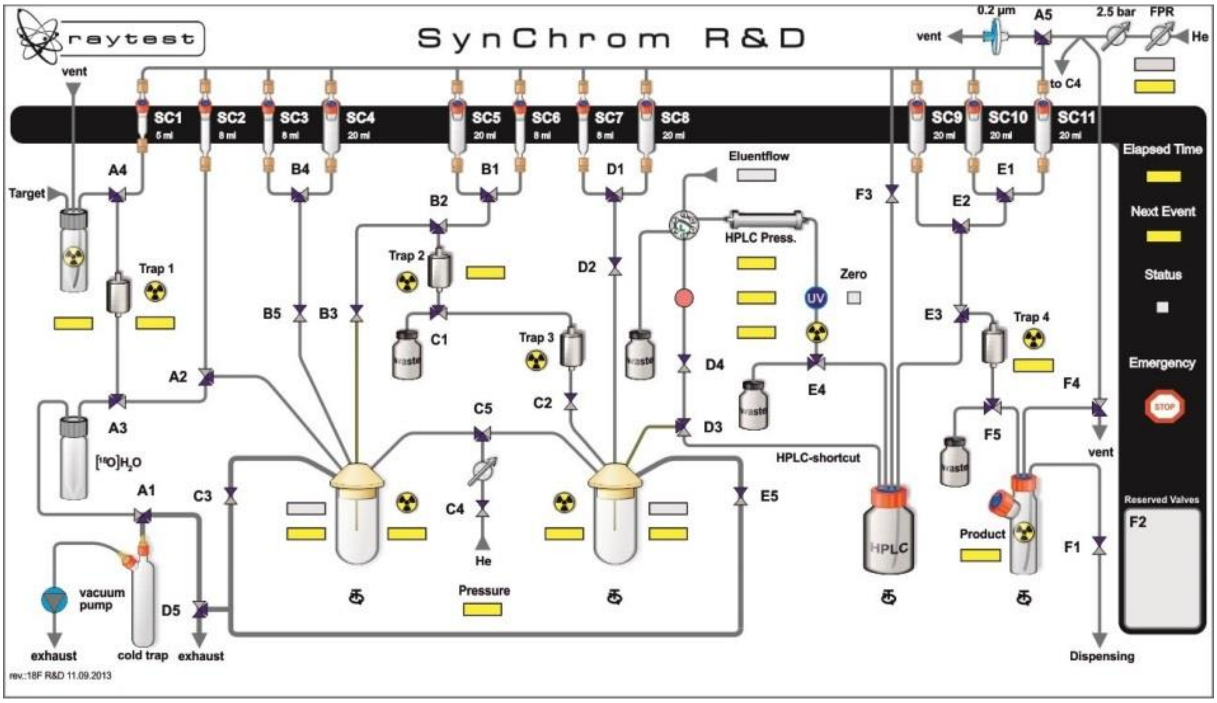

Figure 1: Schematic representation of the setup for the automated radiosynthesis of [^{18}F]**RM273** using the Synchrom R&D EVO III automated synthesizer (Elysia-Raytest, Germany). [^{18}F]Fluoride (4-12 GBq) was trapped on a Waters QMA cartridge (Trap 1) and eluted with a solution (vial SC1) containing 100 µL of $TBAHCO_3$ and 30 µL K_2CO_3 dissolved in a mixture of H_2O/MeCN (1:4, *v/v*) into the reaction vessel and dried via azeotropic distillation. Additional 1.5 mL of dried MeCN was added (vial SC2). After complete dryness, a solution containing 1 mg of boronic acid pinacol ester **45** and 5 mg (7.5 µmol) $Cu(py)_4(OTf)_2$ in DMA/*t*-BUOH (2:1, *v/v*, vial SC3) was added, and the reaction mixture was stirred at 130 °C for 10 min. To remove the excess of copper catalyst and some UV impurities a C18 light cartridge (Trap 2) was applied prior to semi-preparative the HPLC isolation. For that purpose, the reaction mixture was diluted with 18 mL H_2O (vial SC4) and the cartridge eluted with 2.5 mL MeCN (vial SC5). After further dilution with 2.5 mL H_2O (vial SC7), the solution was transferred to the semi-preparative HPLC. [^{18}F]RM273 was collected in the HPLC collection vial containing 40 mL of H_2O and trapped in the Sep-Pak® C18 light cartridge (Trap 4). The cartridge was washed with 2 mL H_2O (vial SC10), and [^{18}F]**RM273** eluted with 1.3 mL EtOH (vial SC11).

3. Quantification of radiometabolites in mouse

Table 1. Radiometabolite analysis of [18F]**RM273** in different tissues 30 min p.i.

	Experiment 1		Experiment 2			
	**Mouse 1**		**Mouse 2**		**Mouse 3**	
	**Extraction efficiency (%)**	**Intact tracer (%)**	**Extraction efficiency (%)**	**Intact tracer (%)**	**Extraction efficiency (%)**	**Intact tracer (%)**
**Plasma**	88	23	85	37	84	28
**Brain**	96	100	96	100	94	100
**Liver**	91	34	91	36	92	28
**Pancreas**	98	100	98	96	98	96
**Bone Marrow**	//	//	98	97	99	97

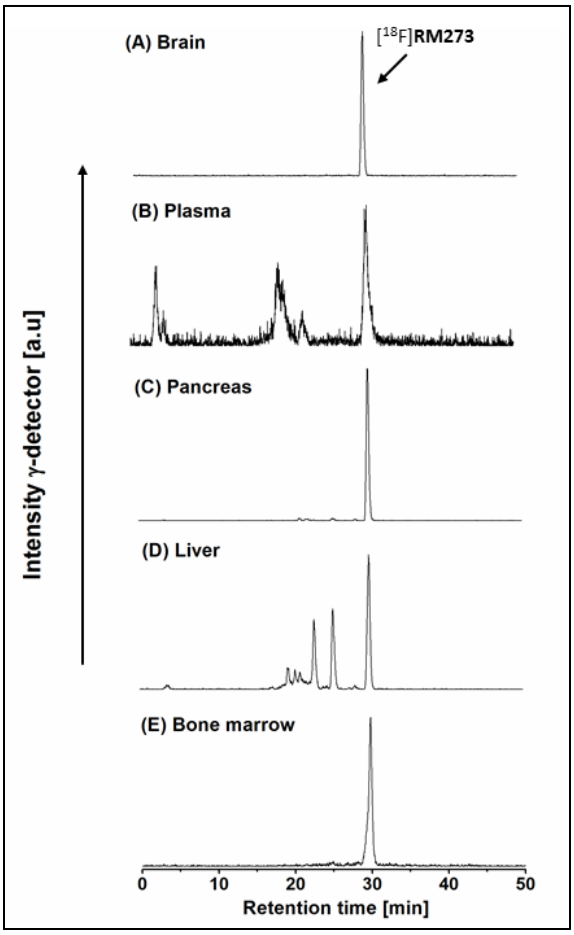

Figure 2. Representative radio-HPLC chromatograms of the metabolism studies of [18F]**RM273** in different tissues obtained at 30 min p.i.. (A) Brain, (B) Plasma, (C) Pancreas, (D) Liver and (E) Bone marrow. Conditions: Reprosil-Pur C18-AQ (250 x 4.6 mm; 5 µm); gradient (eluent A 10% MeCN/20 mM $NH_4OAc_{aq.}$; eluent B 90 % MeCN/20 mM $NH_4OAc_{aq.}$; 0–10 min 100 % A, 10–30 min up to 100 % B, 30–35 min 100 % B, 35-36 min 100 % A, 36–45 min 100 % A); flow: 1 mL·min^{-1}.

4. ¹H NMR spectra of compounds 34-42, 44 and 45

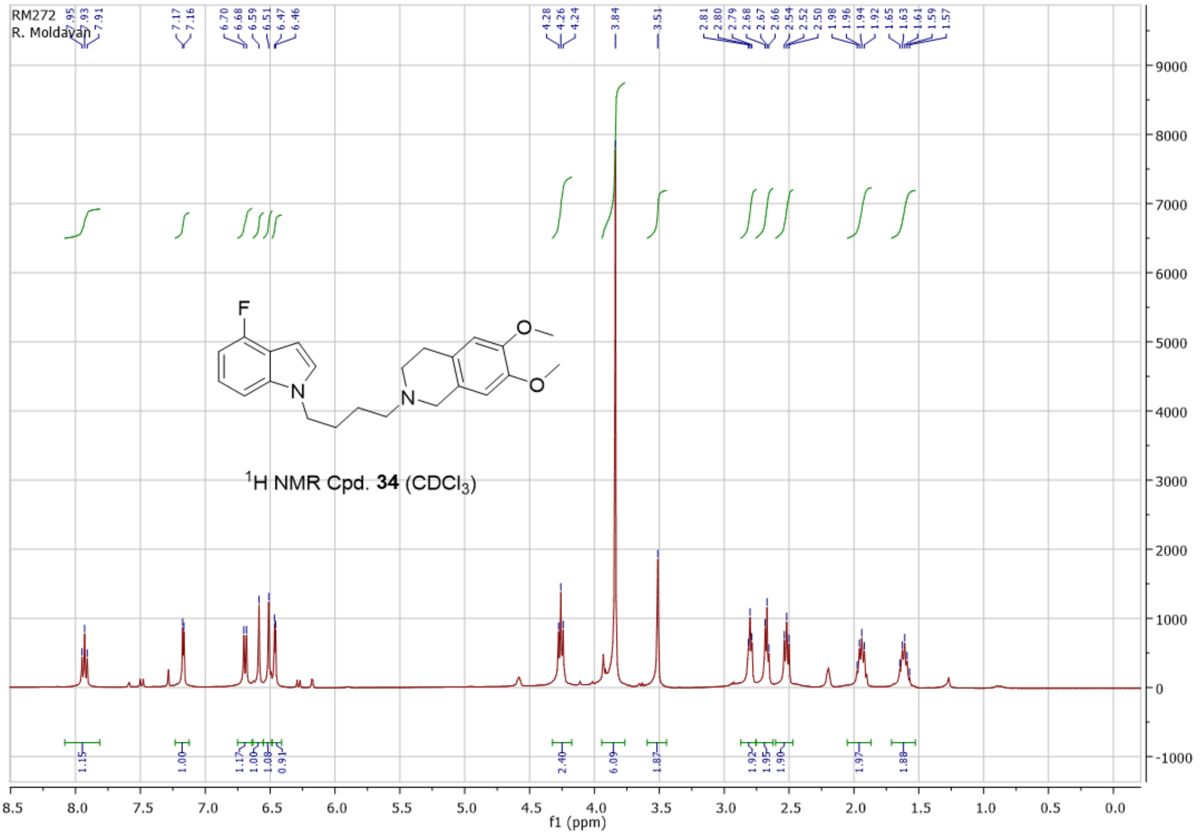

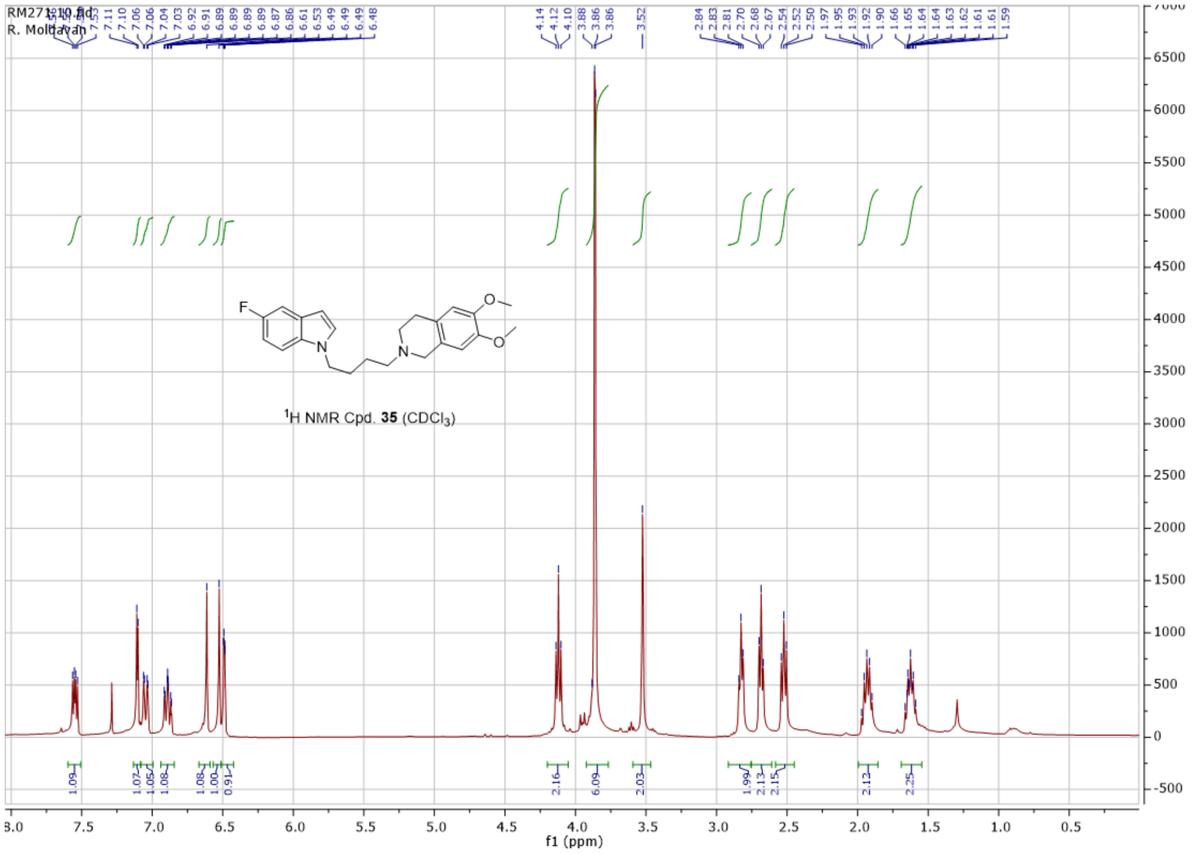

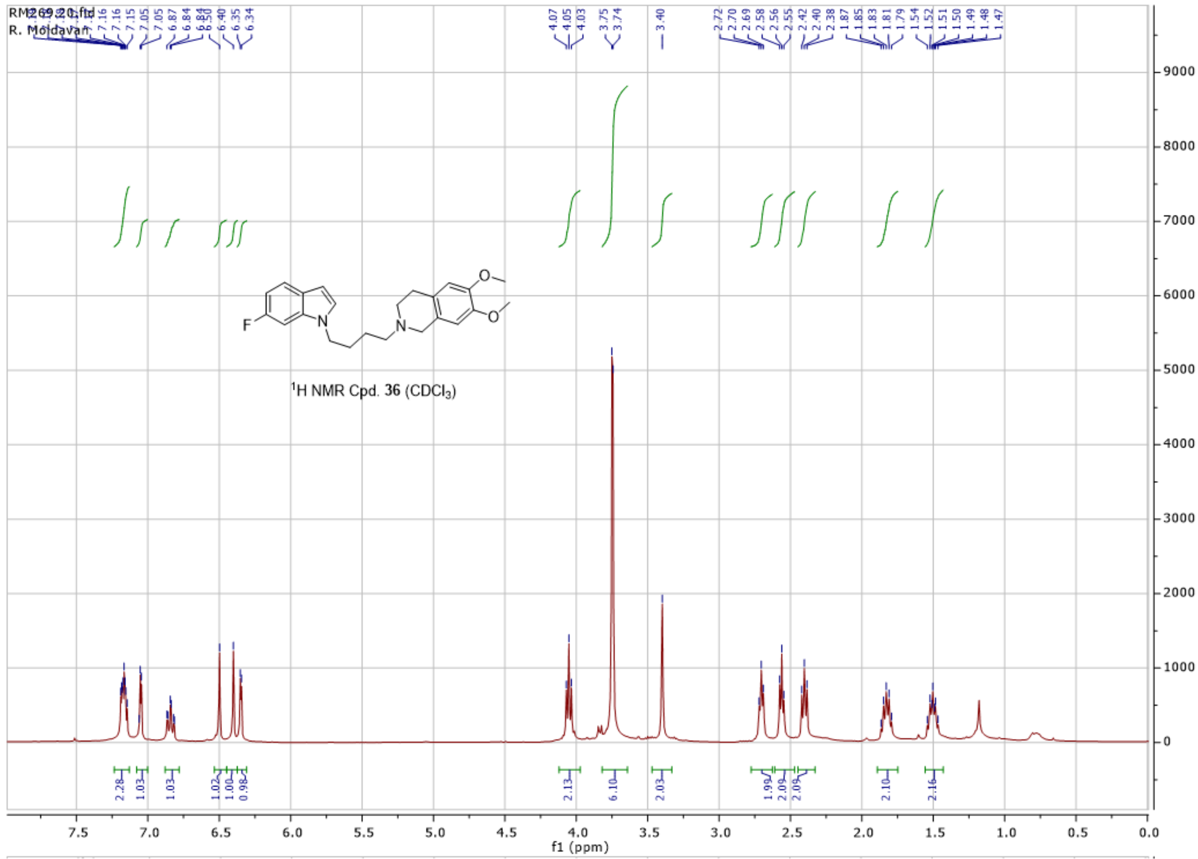
RM269.20.fid
R. Moldavan
F
N
O
¹H NMR Cpd. 36 (CDCl₃)
f1 (ppm)

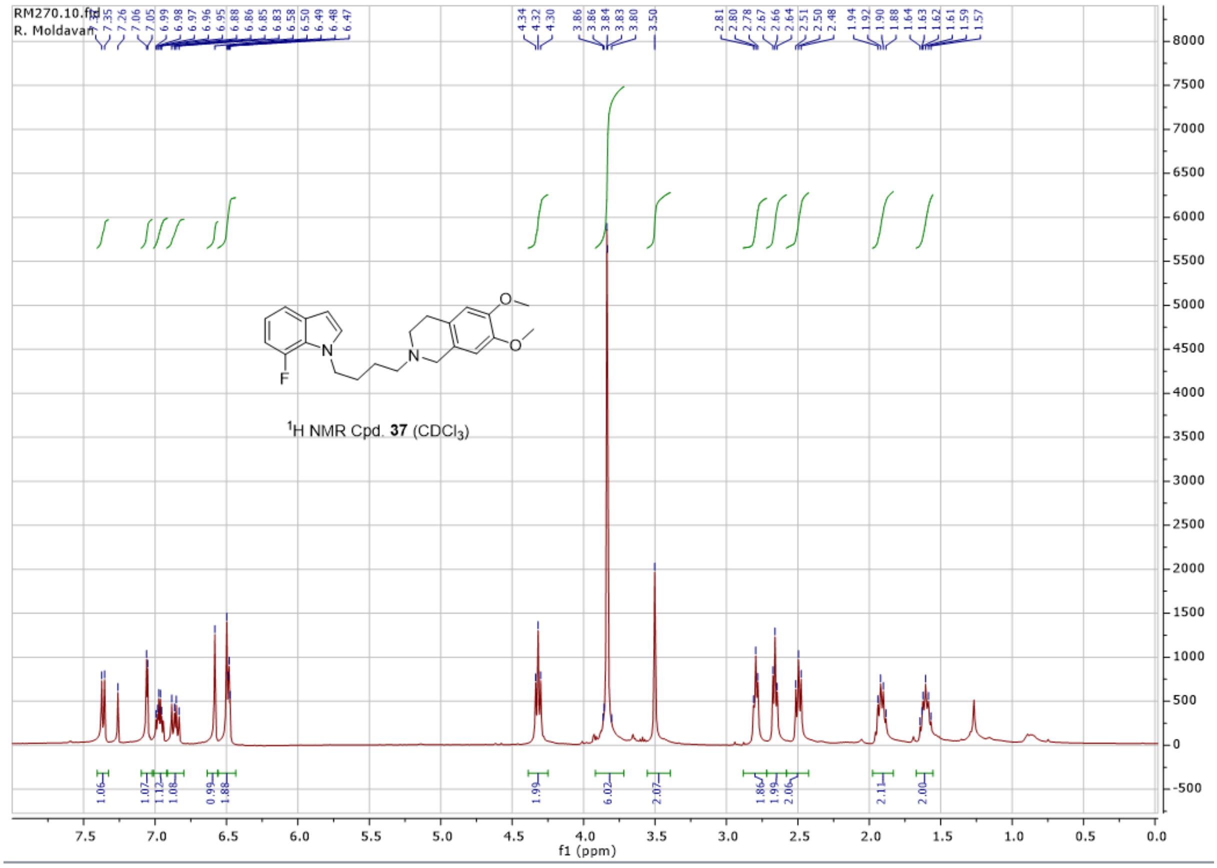
RM270.10.fid
R. Moldavan
F
N
O
¹H NMR Cpd. 37 (CDCl₃)
f1 (ppm)

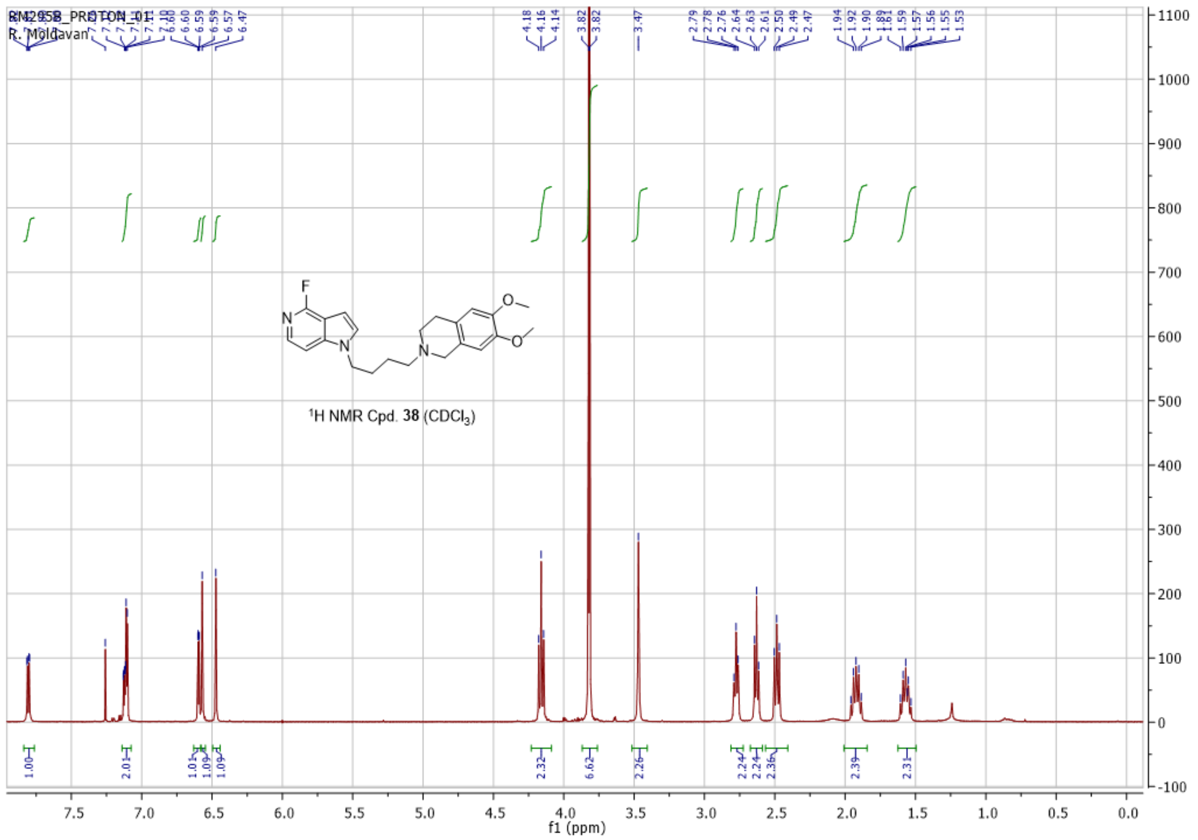

^{1}H NMR Cpd. **38** ($CDCl_3$)

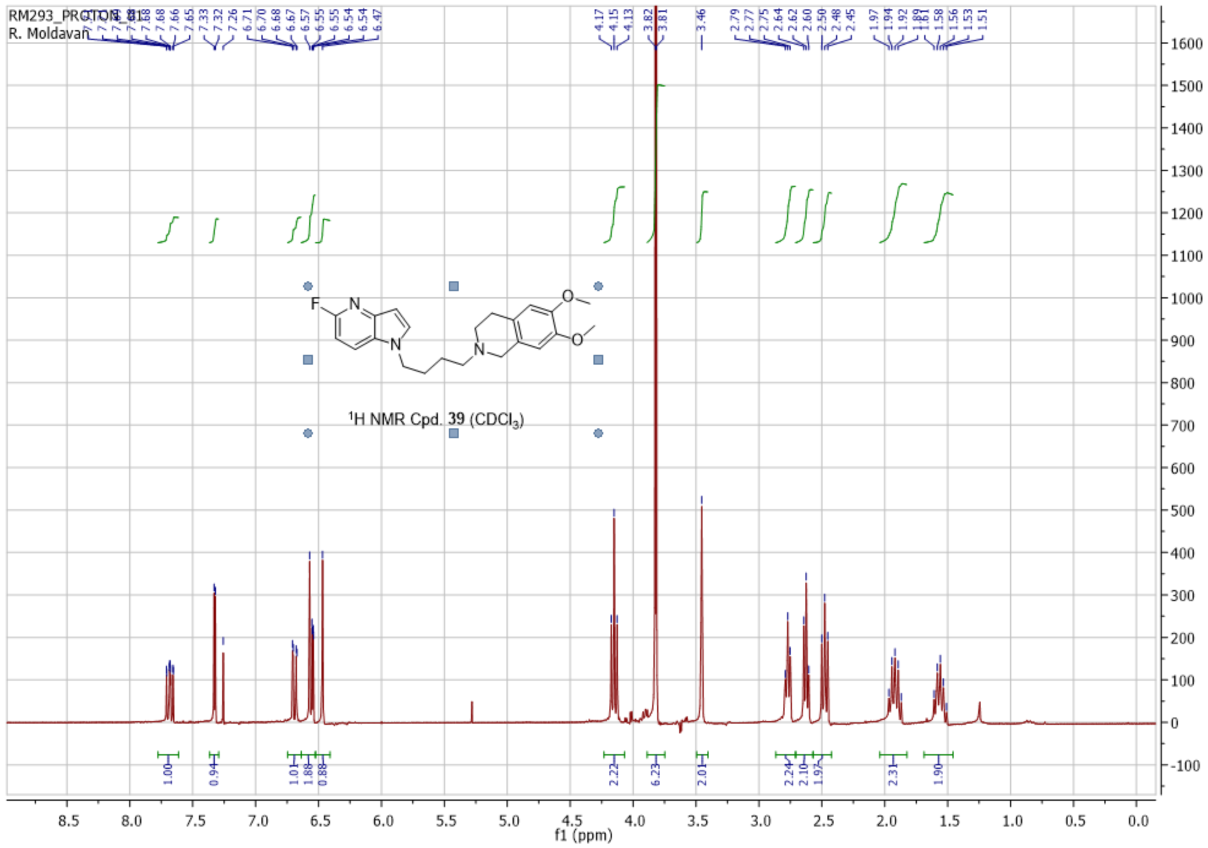

^{1}H NMR Cpd. **39** ($CDCl_3$)

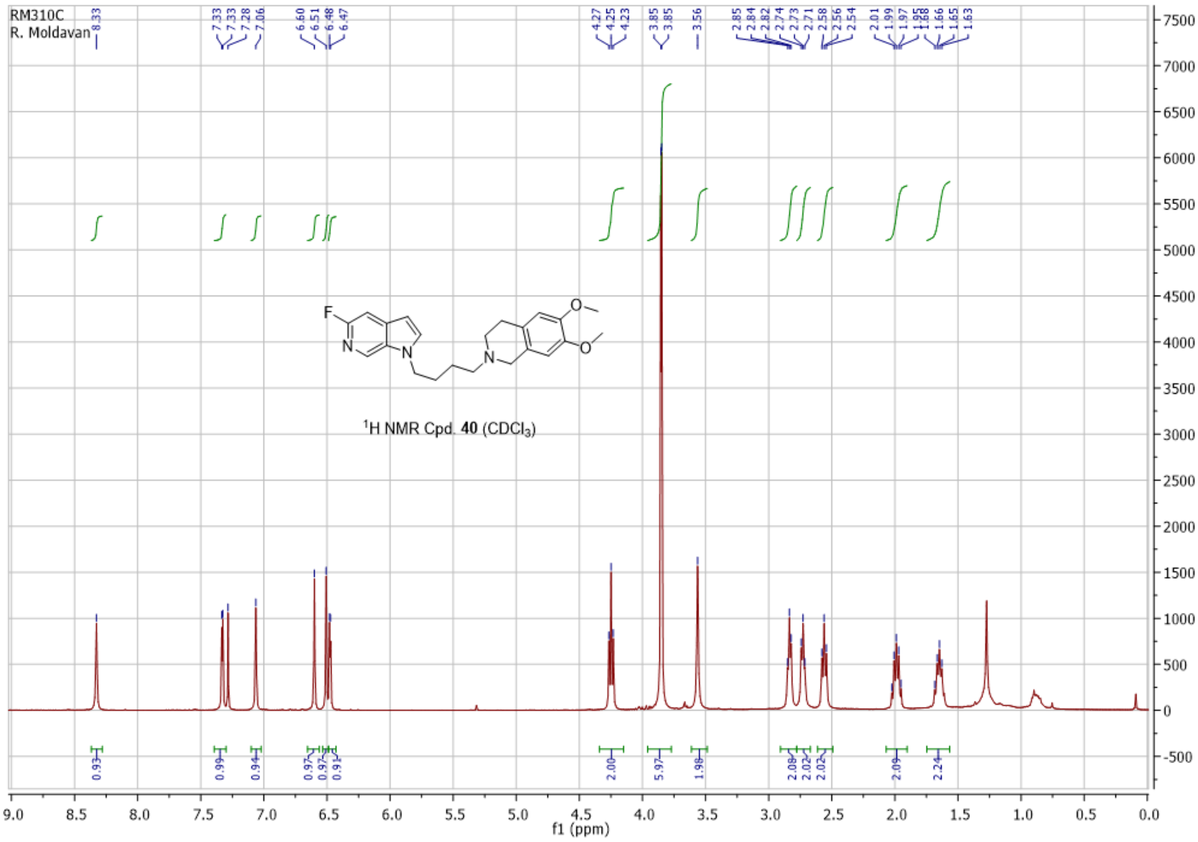
RM310C
R. Moldavan
1H NMR Cpd. 40 (CDCl3)
f1 (ppm)

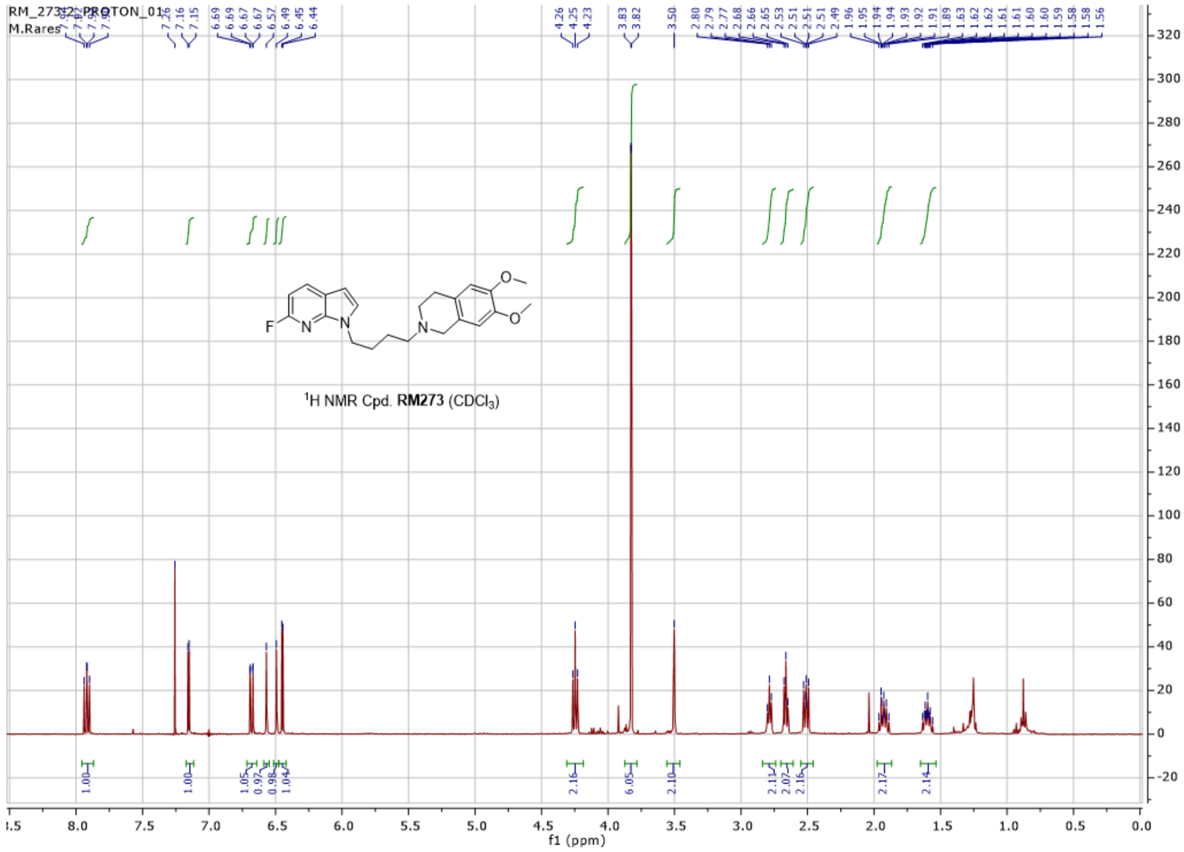
M.Rares
1H NMR Cpd. RM273 (CDCl3)
f1 (ppm)

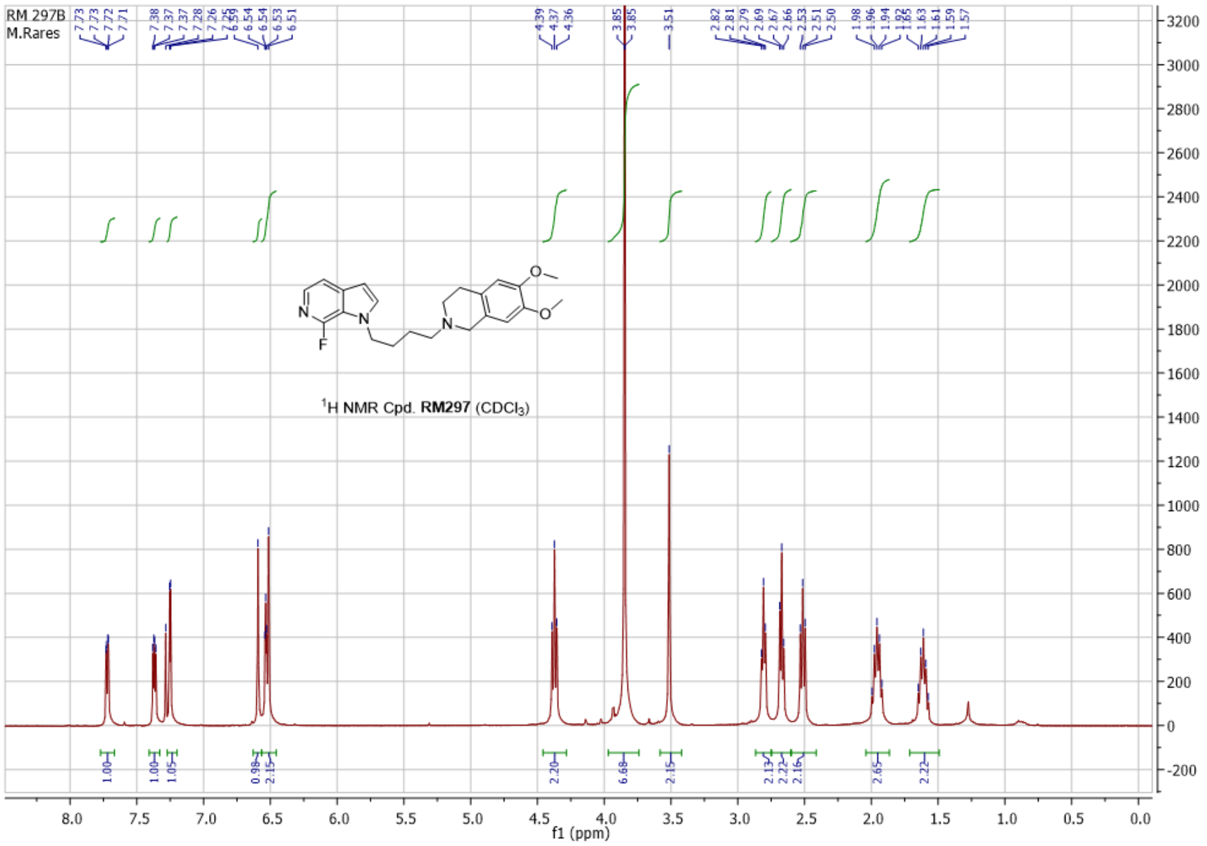
RM 297B
M.Rares
1H NMR Cpd. RM297 (CDCl3)
f1 (ppm)

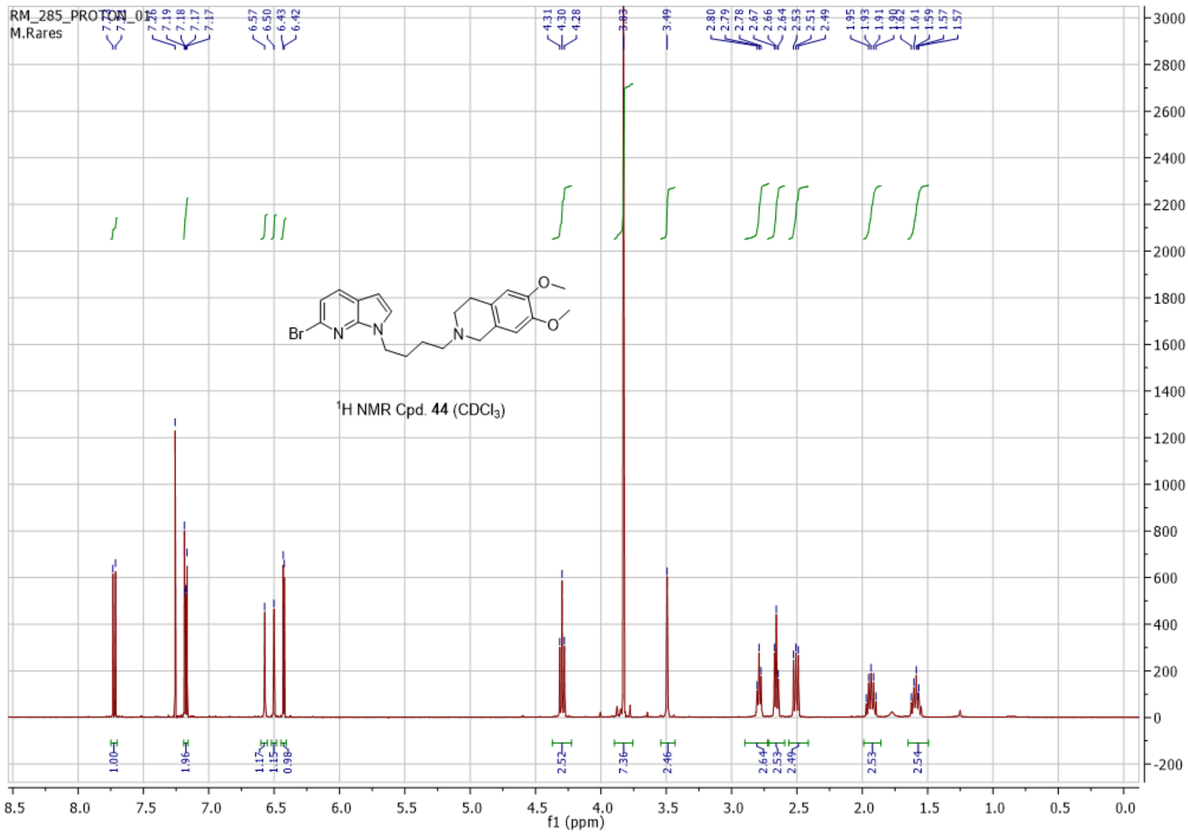
RM_285_PROTON_01
M.Rares
1H NMR Cpd. 44 (CDCl3)
f1 (ppm)

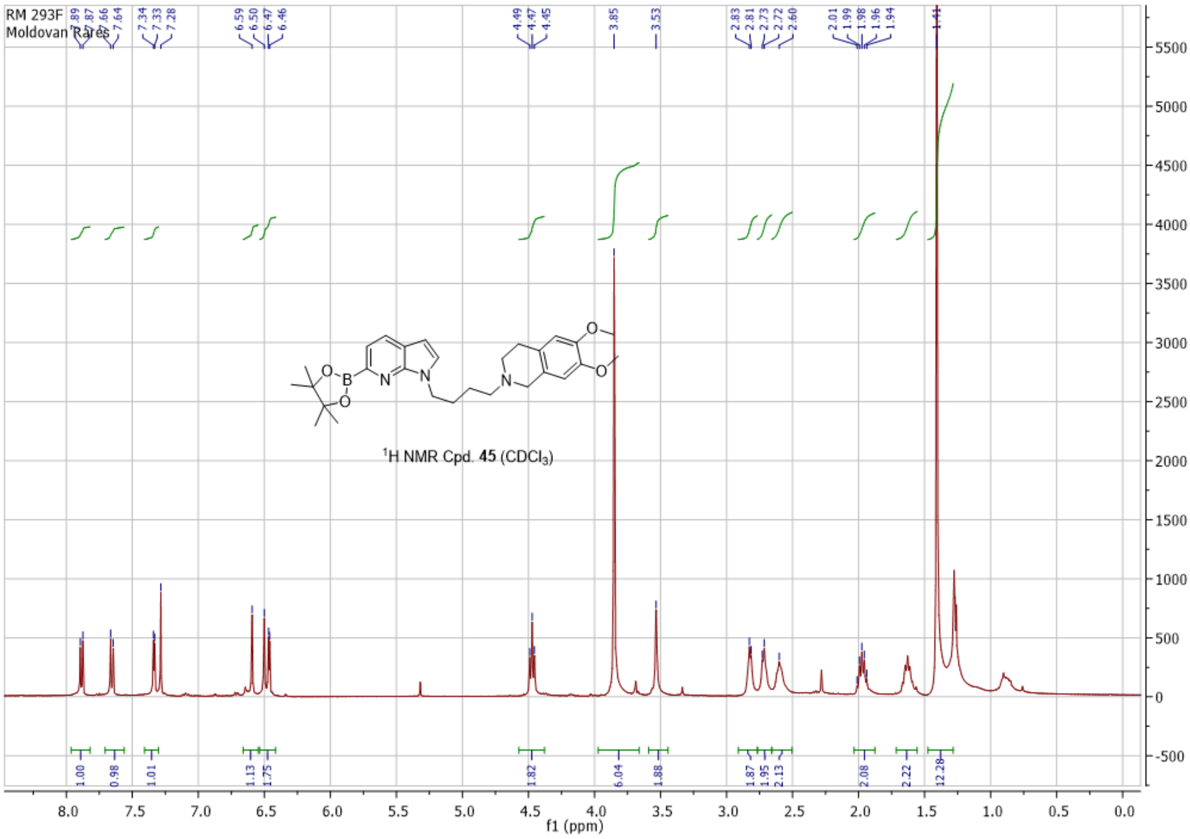
RM 293F
Moldovan Rares
1H NMR Cpd. 45 (CDCl3)
f1 (ppm)
